# Metabolic regulation of ATM-KAT8-RPA1 axis in DNA damage repair and chemoresistance

**DOI:** 10.1016/j.isci.2026.117908

**Published:** 2026-10-26

**Authors:** Xin Ding, Jie Yang, Jinhuan Wu, Mingpeng Jin, Zhe Wang, Xiaoning Yang, Xuanhe Wang, Yifei Song, Bingsong Huang, Rui Li, Yu Li, Yiming He, Wen Zheng, Qianwen Wang, Zhikai Zeng, Yuping Chen, Jian Yuan

**Affiliations:** 1 State Key Laboratory of Cardiology and Research Center for Translational Medicine, Shanghai East Hospital, Tongji University School of Medicine, Shanghai 200120, China; 2 Cancer Center, Tongji University School of Medicine, Shanghai 200331, China; 3 Department of Thoracic Surgery, The First Affiliated Hospital of Nanchang University, Nanchang 330006, China; 4 Institute of Medicinal Biotechnology, Chinese Academy of Medical Sciences & Peking Union Medical College, Beijing 100050, China; 5 Department of Thoracic Surgery, Shanghai Pulmonary Hospital, Tongji University School of Medicine, Shanghai, China; 6 Medical and Radiation Oncology, Department of the Second Affiliated Hospital of Wenzhou Medical University, Wenzhou 325000, China

**Keywords:** DNA damage, homologous recombination, ATM, KAT8, RPA1

## Abstract

Post-translational modifications are recognized as critical regulators of the DNA damage response. In this study, we determined that replication protein A1 (RPA1), a modulator of homologous recombination (HR) repair, is lactylated at lysine 167 (K167) in response to DNA damage. This modification is catalyzed by the histone acetyltransferase KAT8 and is dependent on ATM-mediated phosphorylation of KAT8. Lactylation at K167 enhances the DNA-binding capacity of RPA1, which in turn facilitates HR repair. The inhibition of KAT8 or lactate dehydrogenase (LDH), which increases intracellular lactate level, markedly reduces RPA1 lactylation, impairs HR repair, and increases the sensitivity of tumor cells to chemotherapy in patient-derived xenograft (PDX) models. Furthermore, a cell-penetrating peptide (CPP) that selectively disrupts this modification can sensitize tumors to genotoxic treatment. These findings establish RPA1 lactylation as a critical regulator of HR repair and reveal a potential therapeutic strategy in which this modification can be targeted to overcome chemoresistance.

## Highlights

• RPA1 K167 lactylation promotes its DNA binding and homologous recombination repair
• KAT8 catalyzes RPA1 K167 lactylation in response to DNA damage
• ATM phosphorylation of KAT8 at S392 promotes RPA1 K167 lactylation
• Targeting RPA1 K167 lactylation sensitizes tumors to chemotherapy

## Graphical abstract

**Figure:**
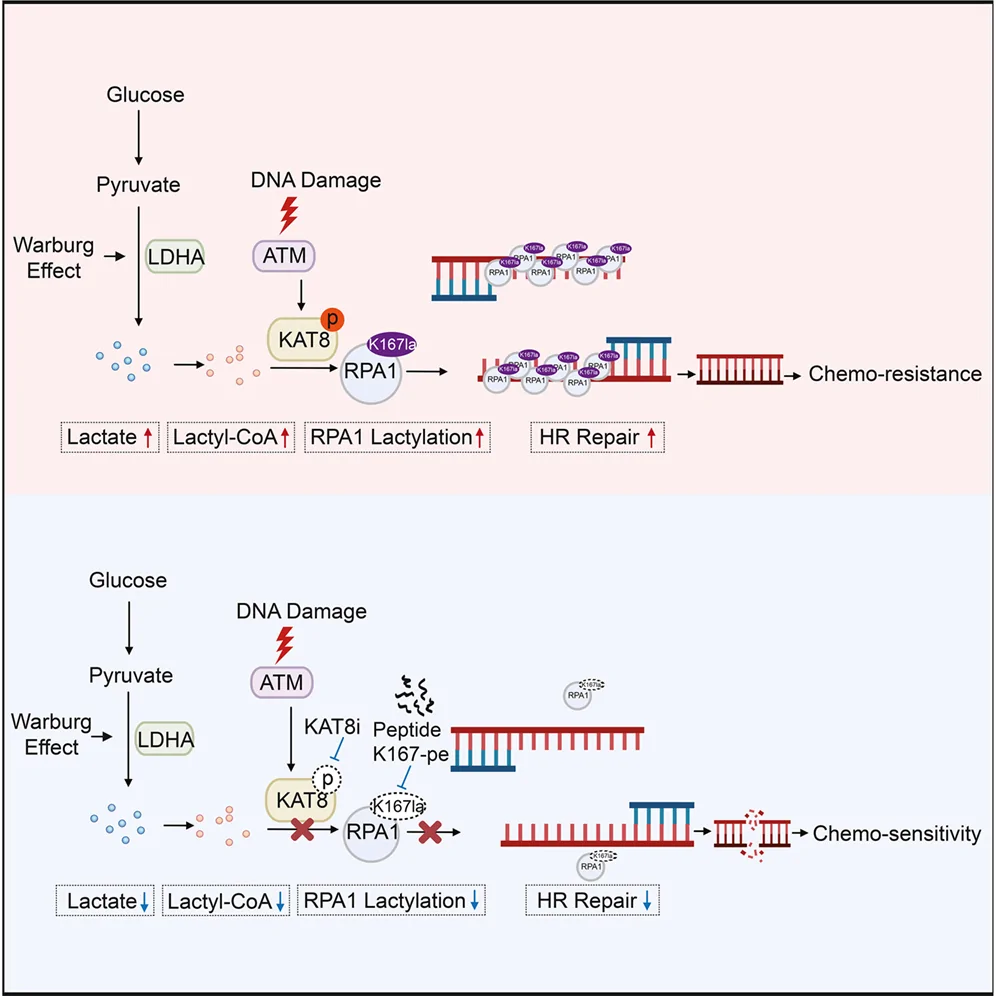

## Teaser

Cancer; Molecular biology

## Introduction

The faithful repair of DNA double-strand breaks (DSBs) is essential for genome stability and cell survival. DSBs represent one of the most cytotoxic forms of DNA damage and can result from replication stress, ionizing radiation, or chemotherapeutic agents.^1^^,^^2^^,^^3^ Homologous recombination (HR) serves as the most accurate mechanism for DSB repair and is primarily active during the S and G2 phases when sister chromatids are available as templates.^4^^,^^5^ HR dysregulation has been implicated in tumorigenesis, cancer progression, and therapeutic resistance.^6^^,^^7^^,^^8^ Upon the occurrence of DNA DSBs, HR repair is initiated. Upon the occurrence of DNA DSBs, MRN complex is recruited to damaged sites to initiate DNA-end resection.^9^^,^^10^ Further resection is extended by EXO1 and DNA2 nucleases to generate 3′-ssDNA overhangs,^11^^,^^12^ which are rapidly coated by RPA to stabilize the single-stranded regions.^13^ BRCA2 then loads RAD51 onto ssDNA by displacing RPA, forming RAD51 filaments essential for homology search and strand invasion.^14^^,^^15^ This facilitates the use of a sister chromatid as a repair template, completing error-free DSB repair.^15^^,^^16^

Replication protein A1 (RPA1), the largest subunit of the heterotrimeric RPA complex, is essential for stabilizing single-stranded DNA during replication and repair.^13^ Loss or mutation of RPA1 impairs DNA DSB repair and leads to genomic instability.^17^^,^^18^ Structurally, RPA1 comprises several oligonucleotide/oligosaccharide-binding (OB) folds: the N-terminal OB-F domain (RPA70N) mediates protein-protein interactions; central OB-A and OB-B domains (RPA70A and RPA70B) directly bind ssDNA; and the C-terminal OB-C domain (RPA70C) supports trimerization and DNA-binding coordination.^19^ Mutations or loss of RPA1 have been linked to genomic instability and are associated with cancer such as breast and ovarian carcinoma, and Fanconi anemia-like phenotypes.^20^^,^^21^^,^^22^ RPA1 is post-translationally modified by phosphorylation, SUMOylation, and ubiquitination. Phosphorylation at the N-terminal Ser/Thr-rich region (e.g., Ser33) by ATR regulates DNA damage checkpoint activation and facilitates repair pathway selection.^13^^,^^23^^,^^24^ SUMOylation at lysine residues promotes recruitment of DNA repair proteins and enhances ATR signaling during replication stress.^13^ The precise modulation of RPA activity is crucial for determining HR fidelity and preventing aberrant repair outcomes such as chromosomal translocations and loss of heterozygosity.^25^^,^^26^^,^^27^

Post-translational modifications (PTMs) of DNA repair factors have emerged as key regulators of repair dynamics. PTMs such as phosphorylation, ubiquitination, and acetylation alter protein stability, localization, and protein-protein interactions during the DNA damage response (DDR).^28^^,^^29^^,^^30^^,^^31^^,^^32^ Recently, lysine lactylation—a metabolically regulated PTM derived from lactate—has gained attention for its roles beyond epigenetic control.^33^

The Warburg effect, a hallmark of cancer metabolism, describes a persistent shift toward aerobic glycolysis and elevated lactate production despite adequate oxygen availability.^34^^,^^35^ This metabolic reprogramming not only promotes tumor proliferation, angiogenesis, and immune evasion but also supplies the intermediate metabolite lactate, which serves as a donor for lactylation.^36^^,^^37^ Recent studies suggest that lactylation extends beyond histones to nonhistone proteins, affecting diverse cellular processes.^38^^,^^39^ MRE11, a critical component of the MRN complex, was recently identified as a target of lactylation, enhancing its DNA binding and HR activity following DSBs.^31^ However, the role of lactylation in regulating other HR factors largely unexplored. Of particular interest is whether RPA1, a key mediator of ssDNA protection and homology search, is subject to such regulation.

Herein, we report that RPA1 undergoes lysine lactylation at K167, catalyzed by the histone acetyltransferase KAT8, in response to DNA damage. This modification is dependent on the ATM-mediated phosphorylation of KAT8 and is required for RPA1 chromatin loading and HR activation. The inhibition of KAT8 or lactate metabolism decreases RPA1 lactylation and sensitizes cancer cells to DNA-damaging agents *in vitro* and in patient-derived xenograft (PDX) models. Furthermore, we designed a cell-penetrating peptide (CPP) that is able to target the lactylation of RPA1 at K167 and impair HR repair, resulting in chemosensitivity. These findings reveal an axis linking the Warburg effect to the DDR machinery and suggest that targeting RPA1 lactylation may be a promising strategy to overcome chemoresistance in HR-proficient tumors.

## Results

### RPA1 is lactylated by KAT8 in response to DNA damage

We discovered that RPA1 underwent lactylation and that sodium lactate (NALA) treatment increased the RPA1 lactylation level without affecting its acetylation, whereas sodium oxamate (lactate dehydrogenase (LDH) inhibitor/LDHi) had the opposite effect (Figure 1A). Moreover, the depletion of LDHA/B, isoforms of LDH, significantly reduced the lactylation level of RPA1 (Figure 1B). We also observed an increase in the lactylation level of RPA1 following exposure to multiple DNA-damaging agents (Figure 1C).

**Figure 1:**
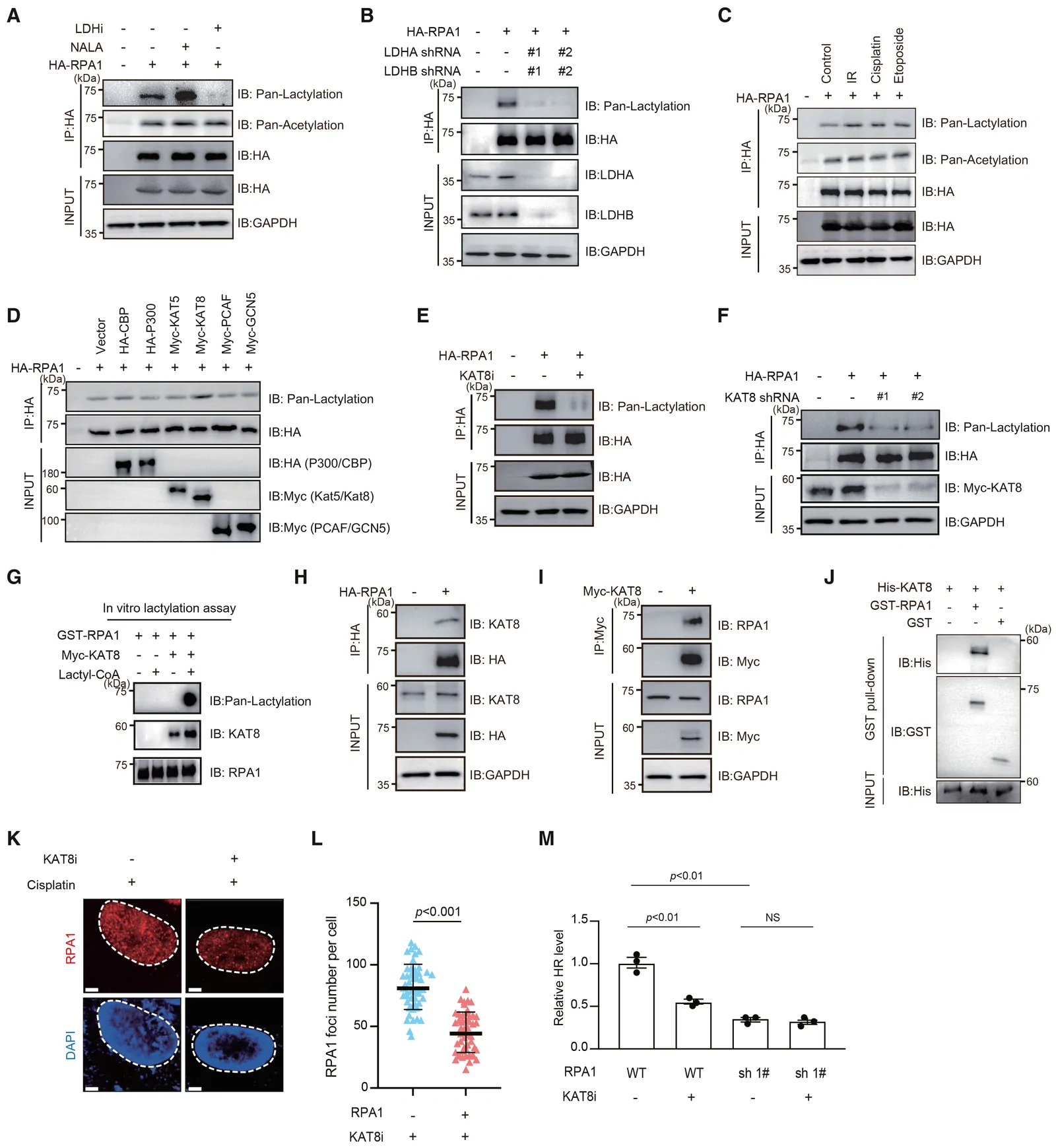
RPA1 is lactylated by KAT8 in response to DNA damage (A) Immunoprecipitation (IP) of HA-tagged RPA1 followed by immunoblotting (IB) with pan-lactylation and pan-acetylation antibodies in HEK293T cells treated with sodium lactate (NALA) or LDH inhibitor (LDHi). (B) HEK-293T cells were transfected with HA-RPA1 and shRNAs targeting LDHA or LDHB. Cell lysates were HA-immunoprecipitated and analyzed by immunoblotting. (C) HEK293T cells expressing HA-RPA1 were treated with ionizing radiation (IR), cisplatin, or etoposide. Cell lysates were HA-immunoprecipitated and analyzed by immunoblotting with the indicated antibodies. (D) HA-RPA1 was co-expressed with indicated acetyltransferase in HEK293T cells. HA immunoprecipitates were analyzed for RPA1 lactylation by immunoblotting. (E and F) HEK293T cells expressing HA-RPA1 were treated with or without a KAT8 inhibitor (E) or with or without KAT8 knockdown (F). HA immunoprecipitates were analyzed by immunoblotting for RPA1 lactylation. (G) Detecting RPA1 lactylation using an *in vitro* lactylation assay on the indicated samples. (H and I) Co-immunoprecipitation assays in HEK293T cells showing the interaction between exogenous HA-RPA1 and endogenous KAT8 (G), and between exogenous Myc-KAT8 and endogenous RPA1 (H). (J) GST pull-down assay showing a direct interaction between KAT8 and RPA1. Purified His-KAT8 was incubated with GST-RPA1 or GST control, followed by GST pull-down and immunoblotting with the indicated antibodies. (K and L) Immunofluorescence analysis of RPA1 foci formation in U2OS cells treated with or without a KAT8 inhibitor after cisplatin treatment (K). RPA1 foci were quantified (L). Scale bars, 4 μm. (M) The HR levels of the carried RPA1 WT or RPA1 knockdown HEK293T cells treated with or without KAT8i for 4 h were detected by FACS. For plasmid transfection experiments, “−” indicates transfection with the corresponding empty vector, which was used as a negative control (A–F, and H–I). Data are presented as mean ± SEM. Statistical significance was assessed by one-way ANOVA followed by Tukey’s multiple-comparison test (M) and unpaired Student’s *t* test (L). ∗*p* < 0.05; ∗∗*p* < 0.01; ∗∗∗*p* < 0.001; NS stands for no significant change. ∗*p* < 0.05; ∗∗*p* < 0.01; ∗∗∗*p* < 0.001; NS stands for no significant change.

To further investigate the mechanism underlying RPA1 lactylation, we screened and identified KAT8 as a potential lactyltransferase for RPA1 lactylation (Figure 1D). Treatment with MG149 (KAT8 inhibitor/KAT8i) (Figure 1E) or KAT8 knockdown (Figure 1F) significantly reduced the lactylation level of RPA1. Additionally, KAT8 catalyzed the lactylation (Figure 1G) but not the acetylation of RPA1 *in vitro* (Figure S1A). Co-immunoprecipitation assays showed that HA-RPA1 interacted with endogenous KAT8 (Figure 1H), and reciprocal immunoprecipitation of Myc-KAT8 also pulled down endogenous RPA1 (Figure 1I). Consistently, an *in vitro* GST-pull-down assay using purified GST-RPA1 and His-KAT8 further confirmed that RPA1 directly binds to KAT8 (Figure 1J). Since members of the sirtuin and HDAC families have been reported to mediate protein delactylation,^40^^,^^41^^,^^42^ we first treated HEK293T cells overexpressing HA-RPA1 protein with nicotinamide (NAM), a broad inhibitor of the sirtuin family, or trichostatin A (TSA), a pan-inhibitor of the HDAC family. We found that NAM treatment significantly enhanced RPA1 lactylation, whereas TSA treatment had no detectable effect, suggesting that RPA1 delactylation mainly depends on sirtuins (Figure S1B). To identify the specific sirtuin responsible, we overexpressed different isoforms of Myc-tagged SIRT proteins in HEK293T cells. We found that only Myc-SIRT6 overexpression significantly reduced RPA1 lactylation, whereas other SIRT isoforms had no effect (Figure S1C). These results indicate that SIRT6 may specifically mediate RPA1 delactylation.

Since the main functions of RPA1 in HR repair are to be recruited to DNA damage sites and to protect single-stranded DNA (ssDNA), we next investigated whether KAT8 inhibition affects RPA1 foci formation. Inhibition of KAT8 reduced RPA1 foci formation upon DNA damage (Figures 1K and 1L), leading to decreased HR repair (Figure 1M). Notably, when RPA1 was knocked down using shRNA, as confirmed by immunoblotting (Figure S1D), KAT8 inhibition treatment did further decreased HR repair efficiency (Figure 1M), indicating that KAT8-mediated HR repair depends on RPA1. Taken together, these findings demonstrate that KAT8 can catalyze the lactylation of RPA1, which might play a crucial role in RPA1 focus formation and HR repair.

### RPA1 is modified by lactylation at K167

To determine the amino acid residues of RPA1 subject to lactylation, we conducted mass spectrometry (MS) analysis. The results identified four potential lactylation sites in RPA1 (K88, K163, K167, and K267), as illustrated in Figures 2A and 2B; Figures S2A–S2C. To validate these candidate sites, each lysine residue was individually mutated to arginine and lactylation levels were assessed by immunoblotting. Among the four mutants, only K167R markedly reduced RPA1 lactylation, whereas K88R, K163R, and K267R had little effect (Figures 2C and 2D), identifying K167 as the major lactylation site in RPA1. *In vitro* experiments using RPA1 and KAT8 also demonstrated that RPA1 K167 undergoes lactylation, and this modification was abolished by the RPA1 K167R mutation (Figure S2D). Notably, we found that lactylation at RPA1 K167 did not affect the acetylation or crotonylation level of RPA1 (Figures S2E), which are PTMs of RPA1 that have been reported previously.^43^^,^^44^^,^^45^

**Figure 2:**
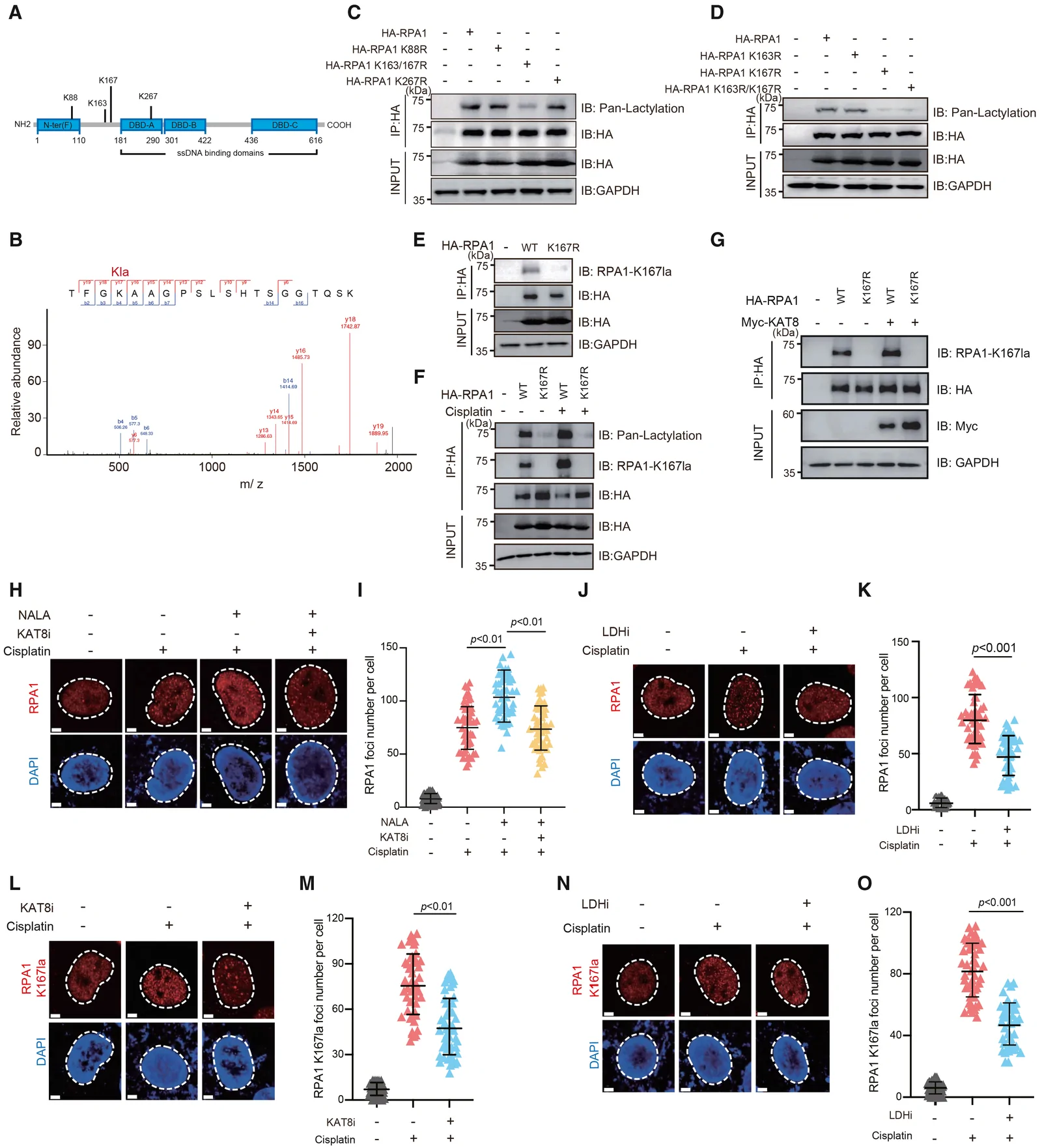
RPA1 is modified by lactylation at K167 (A) Schematic depicting RPA1 key domains and several lysine sites, which were identified to be possibly lactylated by MS were pointed out. (B) Illustration of RPA1 K167 lactylation identified by MS. (C) HA-RPA1 wild type (WT), HA-RPA1 K88R, HA-RPA1 K267R, or an HA-RPA1 K163R/K167R double mutant were expressed in HEK293T cells. HA immunoprecipitates were analyzed by immunoblotting for RPA1 lactylation. (D) HA-RPA1 WT or single mutants (K163R, K167R) and a double mutant (K163R/K167R) were expressed in HEK293T cells, and HA immunoprecipitates were analyzed for RPA1 lactylation by immunoblotting. (E) HA-RPA1 WT and K167R mutant were expressed in HEK293T cells. HA immunoprecipitates were analyzed using the RPA1-K167la-specific antibody. (F) HA-RPA1 WT or HA-RPA1 K167R mutant HEK293T cells were treated with or without cisplatin. HA immunoprecipitates were analyzed for RPA1 lactylation by pan-lactylation and RPA1-K167la-specific antibodies. (G) HEK293T cells overexpressing Myc-KAT8 were transfected with HA-RPA1 WT or K167R mutant. HA immunoprecipitates were analyzed by immunoblotting with RPA1-K167la-specific antibodies. (H and I) Representative picture RPA1 foci in U2OS cells as indicated treatment in upper image (H). RPA1 foci were quantified (I). Scale bars, 4 μm. (J–M) Immunofluorescence images (J and L) and quantification (K and M) of RPA1 foci and RPA1 K167la foci in U2OS cells treated with cisplatin in the presence or absence of LDH inhibitor (LDHi) or KAT8 inhibitor (KAT8i). Scale bars, 4 μm. (N and O) Immunofluorescence images (N) and quantification (O) of RPA1-K167la foci in U2OS cells treated with cisplatin, with or without LDH inhibitor (LDHi). Scale bars, 4 μm. For plasmid transfection experiments, “−” indicates transfection with the corresponding empty vector, which was used as a negative control (C–G). Data are presented as mean ± SEM. Statistical significance was assessed by one-way ANOVA followed by Tukey’s multiple-comparison test (I, K, M, and O). ∗*p* < 0.05; ∗∗*p* < 0.01; ∗∗∗*p* < 0.001; NS stands for no significant change.

To further investigate the lactylation of RPA1 K167, we generated an antibody (termed RPA1-K167la) to specifically recognize RPA1 K167 lactylation, and validated its specificity by immunoblotting (Figure S2F). In Figure 2E, the RPA1-K167la antibody detected a signal in wild-type (WT) RPA1 but not in the RPA1 K167R mutant. Additionally, DNA damage caused by cisplatin (Figure 2F) or overexpression of KAT8 (Figure 2G) significantly increased the lactylation of WT RPA1 but not of the RPA1 K167R mutant. Next, we found that NALA treatment enhanced RPA1 foci formation upon DNA damage and that combined KAT8i or LDHi treatment reversed this effect (Figures 2H–2K). Additionally, we found that KAT8i or LDHi treatment also led to a reduction in RPA1-K167la foci formation in response to DNA damage (Figures 2L–2O). Together, these findings demonstrated that K167 is a major lactylation site in RPA1 in response to DNA damage.

### RPA1 K167 lactylation strengthens the ability of RPA1 to bind DNA

Given that manipulating RPA1 lactylation with KAT8i, LDHi or NALA affects RPA1 focus formation and HR repair, we sought to determine whether this effect is dependent on lactylation at the K167 site of RPA1. Notably, mutation of K167 markedly impaired RPA1 foci formation following DNA damage and abolished the reduction in foci formation caused by LDHi treatment, suggesting that K167 lactylation is required for efficient recruitment of RPA1 to DNA damage sites (Figures 3A and 3B). Consistently, RPA1 K167R exhibited reduced chromatin association, further supporting a role for K167 lactylation in promoting RPA1 chromatin loading (Figure 3C). Moreover, the depletion of KAT8 markedly compromised the recruitment of RPA1 to DNA (Figure 3D). To further directly assess whether RPA1 K167 lactylation affects its DNA-binding ability, we performed an *in vitro* electrophoretic mobility shift assay (EMSA) using purified HA-RPA1 WT and HA-RPA1 K167R proteins. Western blot analysis confirmed that K167 lactylation was markedly reduced in the RPA1 K167R mutant compared with RPA1 WT (Figure 3E). Notably, EMSA showed that RPA1 K167R displayed weaker DNA-binding activity than RPA1 WT, as indicated by reduced protein-DNA complex formation (Figure 3F). To further determine whether K167 lactylation contributes to the recruitment of RPA1 to DNA damage sites in cells, we performed laser micro-irradiation assays using U2OS cells expressing GFP-tagged RPA1 WT or RPA1 K167R. Following laser-induced DNA damage, the accumulation of RPA1 at damage stripes was monitored by live-cell imaging over time. As shown in Figures 3G and 3H, RPA1 WT was rapidly recruited to DNA damage sites, whereas the recruitment of RPA1 K167R was markedly impaired. Collectively, these findings indicate that K167 lactylation promotes both the DNA-binding activity and damage-site recruitment of RPA1.

**Figure 3:**
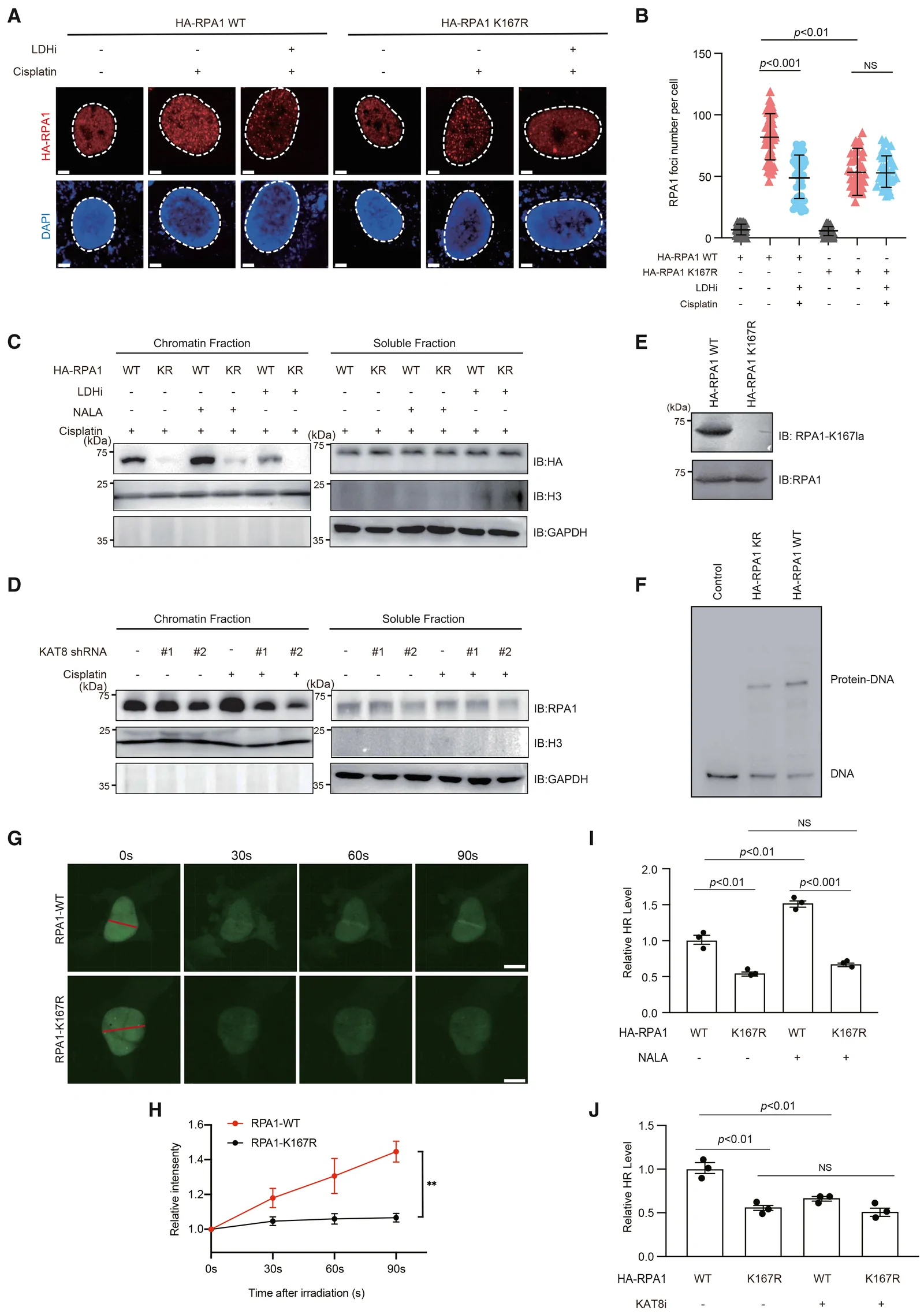
RPA1 lactylation strengthens the ability of RPA1 to bind DNA (A and B) Immunofluorescence images (A) and quantification (B) of HA-RPA1 foci in RPA1 WT and RPA1 K167R U2OS cells treated with Cisplatin, with or without LDH inhibitor (LDHi). Scale bars, 4 μm. (C) HEK293T cells expressing WT HA-RPA1 or HA-RPA1-K167R were pretreated with LDHi or NALA for 24 h, followed by cisplatin stimulation to induce DNA damage. Chromatin and soluble fractions were then isolated and analyzed by immunoblotting to examine HA-RPA1 recruitment. (D) HEK293T cells with or without KAT8 knockdown were treated with or without cisplatin stimulation. Chromatin and soluble fractions were then isolated and analyzed by immunoblotting to assess RPA1 recruitment. (E) Immunoblot analysis of RPA1 K167 lactylation in HEK293T cells expressing HA-RPA1 WT or HA-RPA1 K167R. (F) Electrophoretic mobility shift assay (EMSA) showing the DNA-binding ability of purified HA-RPA1 WT and HA-RPA1 K167R proteins. (G and H) Laser micro-irradiation assay showing the recruitment of GFP-tagged RPA1 WT and RPA1 K167R to DNA damage sites in U2OS cells. Representative live-cell images were acquired at the indicated time points (0, 30, 60, and 90 s) following laser-induced DNA damage (G), and quantification of the relative fluorescence intensity at damage stripes is shown (H). Scale bars, 10 μm. (I) The HR levels of the carried RPA1 WT or RPA1 K167R HEK293T cells treated with or without NALA for 24 h were detected by FACS. (J) The HR levels of the carried RPA1 WT or RPA1 K167R HEK293T cells treated with or without KAT8i for 4 h were detected by FACS. Data are presented as mean ± SEM. Statistical significance was assessed by one-way ANOVA followed by Tukey’s multiple-comparison test (B, I, and J) or two-way ANOVA (H). ∗*p* < 0.05; ∗∗*p* < 0.01; ∗∗∗*p* < 0.001; NS stands for no significant change.

Given the essential role of RPA1 in HR repair, we explored the potential regulatory function of RPA1 K167 lactylation in HR repair. As shown in Figure 3I, RPA1 K167R significantly reduced HR repair efficiency compared to RPA1 WT. Treatment with NALA enhanced HR repair in the presence of RPA1 WT but had no effect in the presence of RPA1 K167R. Conversely, the inhibition of KAT8 suppressed HR repair in the presence of RPA1 WT but did not further affect HR repair efficiency in the presence of RPA1 K167R (Figure 3J). Since the replacement of RPA by RAD51 represents a key step in HR,^46^ we next examined whether RPA1 K167 lactylation influences this process. Following cisplatin-induced DNA damage, chromatin fractionation assays revealed that HEK293T cells expressing the K167R mutant displayed reduced chromatin accumulation of RPA1 and impaired recruitment of RAD51 compared with cells expressing wild-type RPA1. These results suggest that RPA1 K167 lactylation may promote the efficient replacement of RPA by RAD51 during HR (Figure S3A). Together, our results revealed that RPA1 K167 lactylation promote chromatin recruitment and enhance HR repair.

### The ATM-mediated phosphorylation of KAT8 promoted RPA1 foci formation upon DNA damage

Ataxia telangiectasia mutated (ATM) is a central kinase in the DDR, which phosphorylates multiple DNA repair factors at serine or threonine residues followed by glutamine (SQ/TQ motifs) to coordinate repair. Given its pivotal role and the RPA1 lactylation was elevated following DNA damage, we investigated whether ATM regulates the KAT8-RPA1-HR pathway.^47^ We found that DNA damage induced by Cisplatin markedly enhanced the interaction between RPA1 and KAT8, whereas the inhibition of ATM reversed this effect (Figure 4A). In contrast, inhibition of DNA-PKcs or ATR, which recognize similar SQ/TQ motifs as ATM, did not affect the KAT8-RPA1 interaction (Figure S4A). After analyzing the amino acid sequences of KAT8 and integrating the information from PhosphoSite Plus, we found that two sites on KAT8, serine 386 and 392, conform to the consensus motif for ATR/ATM substrates and are potential phosphorylation sites. Moreover, using an anti-p-SQ/TQ substrate antibody, which detects the phosphorylation of serine (S) or threonine (T) residues followed by glutamine (Q) at the +1 position—a motif specifically phosphorylated by ATM/ATR kinase—we found KAT8 is phosphorylated following DNA damage and that KU-55933 (ATM inhibitor/ATMi) treatment can abolish phosphorylation, indicating that ATM is able to phosphorylate KAT8 (Figure 4B). To further investigate the specific phosphorylation site of KAT8, we generated point mutants at S386 (KAT8 S386A) and S392 (KAT8 S392A). The results showed that mutation of S392 abolished DNA damage-induced phosphorylation of KAT8 detected by the anti-p-SQ/TQ substrate antibody, whereas mutation of S386 had no effect, suggesting that S392 is the primary ATM-mediated phosphorylation site in KAT8 after DNA damage (Figure 4C). Furthermore, *in vitro* kinase assays further confirmed that ATM phosphorylates KAT8 in an S392-dependent manner (Figure S4B). Notably, *in vivo* experiments confirmed that ATM associates with KAT8, and this interaction is enhanced upon DNA damage (Figure S4C).

**Figure 4:**
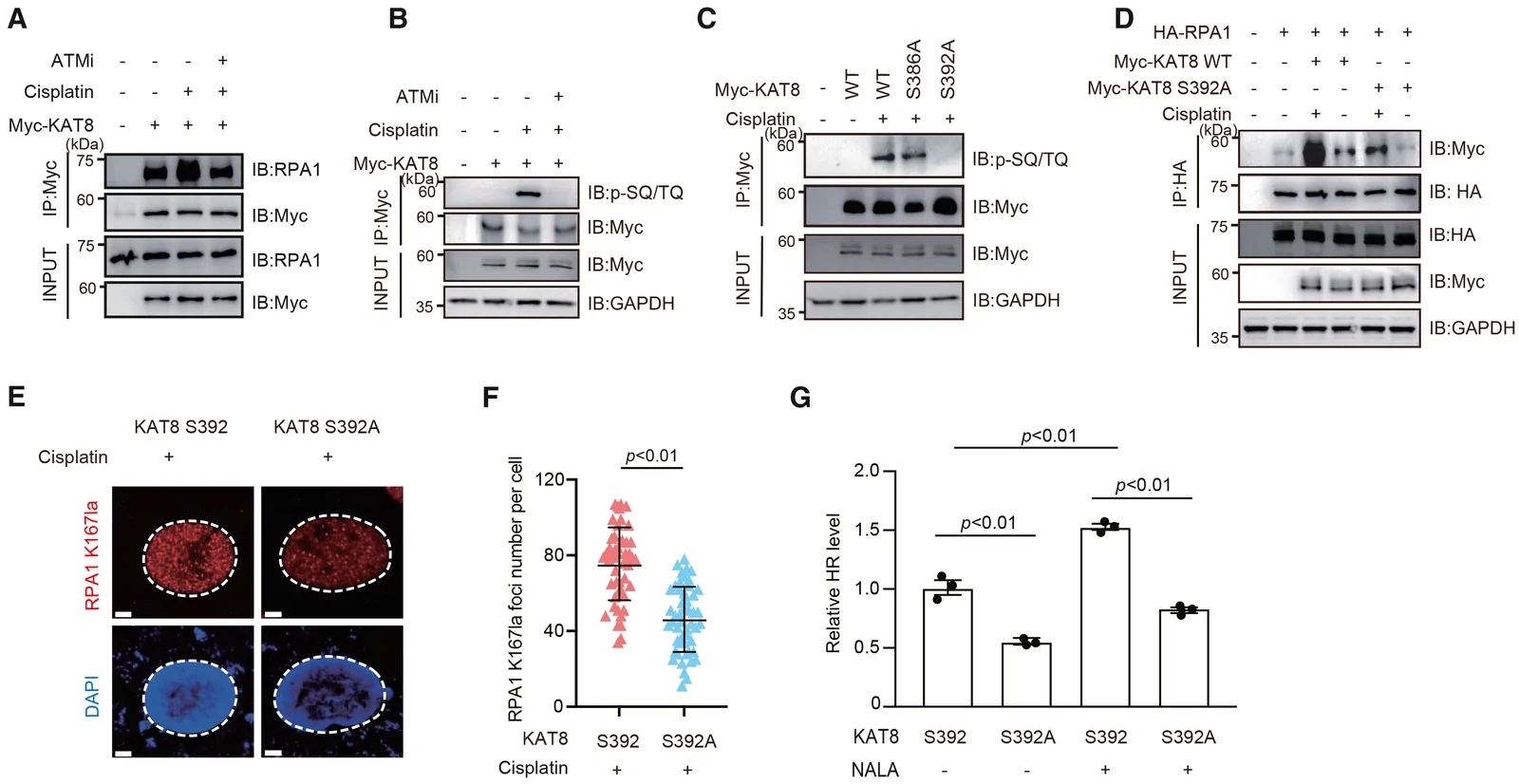
The ATM-mediated phosphorylation of KAT8 promoted the lactylation of RPA1 upon DNA damage (A) HEK293T cells overexpressing Myc-KAT8 were treated with cislpatin and/or ATMi. Myc-KAT8 was immunoprecipitated, and co-precipitated endogenous RPA1 was detected by immunoblotting. (B) HEK293T cells overexpressing Myc-KAT8 were treated with cislpatin and/or ATMi. Myc-KAT8 was immunoprecipitated, and co-precipitated endogenous p-SQ/TQ was detected by immunoblotting. (C) HEK293T cells overexpressing Myc-KAT8 WT, Myc-KAT8 S386A, or Myc-KAT8 S392A were treated with cisplatin. Myc-KAT8 was immunoprecipitated, and phosphorylation levels were analyzed by immunoblotting using the p-SQ/TQ antibody. (D) HEK293T cells overexpressing HA-RAP1 with Myc-KAT8 WT or Myc-KAT8 S392A were treated with Cisplatin. HA-RPA1 was immunoprecipitated, and the interaction with Myc-KAT8 was analyzed by immunoblotting. (E and F) Immunofluorescence images (E) and quantification (F) of RPA1 K167la foci in KAT8 knockdown U2OS cells transfected with KAT8 WT or S392A mutant for 48 h, followed by cisplatin treatment. Scale bars, 4 μm. (G) The HR levels of the carried KAT8 WT or KAT8 S392A HEK293T cells treated with or without NALA for 24 h were detected by FACS. For plasmid transfection experiments, “−” indicates transfection with the corresponding empty vector, which was used as a negative control (A–D). Data are presented as mean ± SEM. Statistical significance was assessed by one-way ANOVA followed by Tukey’s multiple-comparison test (G) and unpaired Student’s *t* test (F). ∗*p* < 0.05; ∗∗*p* < 0.01; ∗∗∗*p* < 0.001; NS stands for no significant change.

KAT8 S392A (mutation of KAT8 S392) significantly reduced the interaction between KAT8 and RPA1 in co-immunoprecipitation assays in HEK293T cells co-expressing HA-RPA1 and Myc-KAT8 WT or S392A, indicating that the ATM-dependent phosphorylation of KAT8 S392 is crucial for its binding to RPA1 (Figure 4D). Furthermore, the KAT8 S392A impaired RPA1 K167la foci formation following DNA damage induced by cisplatin treatment (Figures 4E and 4F). Additionally, compared with KAT8 WT (KAT8 S392), KAT8 S392A obviously reduced HR repair efficiency, even under NALA treatment conditions (Figure 4G). In summary, the ATM-mediated phosphorylation of KAT8 at S392 is essential for its interaction with RPA1, promoting RPA1 foci formation and enhancing HR repair following DNA damage.

### RPA1 lactylation enhances cancer cell resistance to chemotherapy

Given that RPA1 lactylation promotes HR repair and that high DNA repair activation usually leads to chemoresistance,^48^^,^^49^ we next investigated whether RPA1 lactylation influences the chemoresponse in cancer cells. Because HR-deficient cells are particularly sensitive to PARP inhibitors,^50^^,^^51^ we selected olaparib, a clinically approved PARP inhibitor widely used in breast cancer treatment,^52^ to evaluate the impact of RPA1 lactylation on therapeutic response. We first examined RPA1 lactylation levels in MCF 10A (used as a normal control) and various breast cancer cell lines, with relatively higher levels observed in Hs578T and HCC1806 cells and a more modest increase in SUM-159 cells (Figure 5A). Then, we observed that LDHi or KAT8i treatment sensitized Hs578T, HCC1806, and SUM-159 cells to olaparib (Figures 5B–5D and S5A–S5C), whereas NALA treatment rendered SUM-159 cells resistant to olaparib (Figures 5E and S5D). Furthermore, compared with RPA1 WT cells, RPA1 K167R mutant cells exhibited greater sensitivity to olaparib (Figures 5F, 5G, S5E, and S5F). Notably, LDHi or KAT8i treatment increased the sensitivity of RPA1 WT cells to olaparib, but failed to further sensitize RPA1 K167R mutant cells, suggesting that the ability of LDHi or KAT8i to enhance cancer cell sensitivity to olaparib depends on RPA1 K167 lactylation (Figures 5F, 5G, S5E, and S5F). These findings suggest that RPA1 lactylation plays a key role in the chemoresponse of cancer cells.

**Figure 5:**
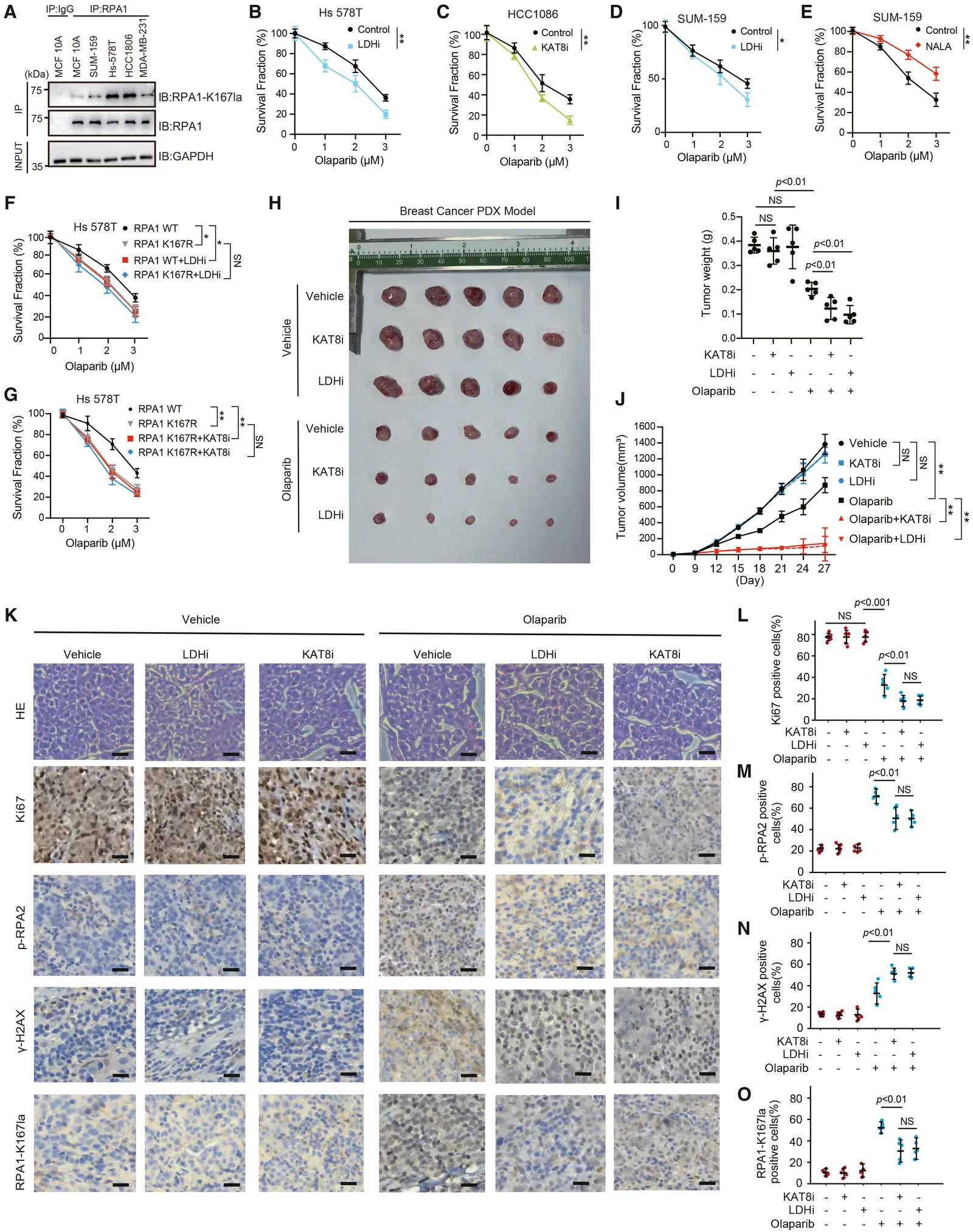
RPA1 lactylation enhances cancer cell resistance to chemotherapy (A) The lactylation levels of RPA1 in various breast cancer cell lines as indicated, analyzed by immunoprecipitation and immunoblotting. (B–E) Cell viability of the indicated breast cancer cell lines treated with LDHi (B and D), KAT8i (C), or NALA (E). (F and G) Cell viability of HS-578T cells expressing RPA1 WT or RPA1 K167R following olaparib treatment in the presence or absence of LDHi (F) or KAT8i (E). (H and I) Tumor images (H) from a breast cancer PDX model treated with vehicle, LDHi, KAT8i, or Olaparib, alone or in combination and quantification of tumor weight (I). (J) Tumor volume measurements over time in the breast cancer PDX model under different treatments. (K–O) H&E staining and Immunohistochemistry analysis of Ki67, p-RPA2, γ-H2AX, and RPA1-K167la in tumor sections from breast cancer PDX models treated with vehicle, LDHi, KAT8i, or olaparib, alone or in combination (K). Quantification of Ki67 (L), p-RPA2 (M), γ-H2AX (N), and RPA1-K167la (O) positive cells. Scale bars, 50 μm. For plasmid transfection experiments, “−” indicates transfection with the corresponding empty vector, which was used as a negative control (A–F, H, and I). Data are presented as mean ± SEM. Statistical significance was assessed by one-way ANOVA followed by Tukey’s multiple-comparison test (I and L–O) and two-way ANOVA (B–G and J). ∗*p* < 0.05; ∗∗*p* < 0.01; ∗∗∗*p* < 0.001; NS stands for no significant change.

To further verify the role of RPA1 lactylation in the chemoresponse of breast cancer *in vivo*, a breast cancer PDX model was used to perform cancer killing assays. As RAD51 foci formation is a hallmark of HR activity, robust RAD51 foci formation was observed in tumor-derived cells following cisplatin-induced DNA damage (Figure S5G), indicating that this PDX model was HR proficient (HR-proficient). As shown in Figures 5H–5J, treatment with KAT8i or LDHi enhanced the antitumor efficacy of olaparib. In addition, in the olaparib-treated groups, both KAT8i and LDHi markedly reduced the staining intensities of phospho-RPA2 (p-RPA2 is a well-established marker associated with RPA complex engagement and HR repair, as RPA2 is phosphorylated following RPA binding to single-stranded DNA53^13^^,^^53^^,^^54^), Ki67, and RPA1-K167la while increasing the staining intensity of γ-H2AX, indicating that KAT8i and LDHi impaired DNA damage repair *in vivo* and thus sensitized tumors to chemotherapy (Figures 5K–5O). Collectively, these findings suggest that combining KAT8i or LDHi with chemotherapy may represent a promising therapeutic strategy for tumors.

### Peptidic inhibition of RPA1 K167 lactylation enhances chemosensitivity

On the basis of the previous findings, we propose that high levels of RPA1 K167 lactylation activate HR repair and result in chemoresistance, indicating a strategy that specifically targets RPA1 K167 lactylation is needed for cancer therapy. Based on motifs involved in protein PTMs, specifically generated peptides have been shown to be a promising and effective strategy to inhibit protein’s PTMs. After analyzing the sequence around the RPA1 K167 site, we synthesized six short peptides fused with CPPs, which have become one of the most popular and effective tools for the intracellular delivery of biomolecules^31^^,^^55^^,^^56^ (Figure 6A). Among these peptides, K167-peptide-6# (referred to as K167-pe) exhibited the most potent inhibitory effect against RPA1 lactylation (Figures 6B and 6C).

**Figure 6:**
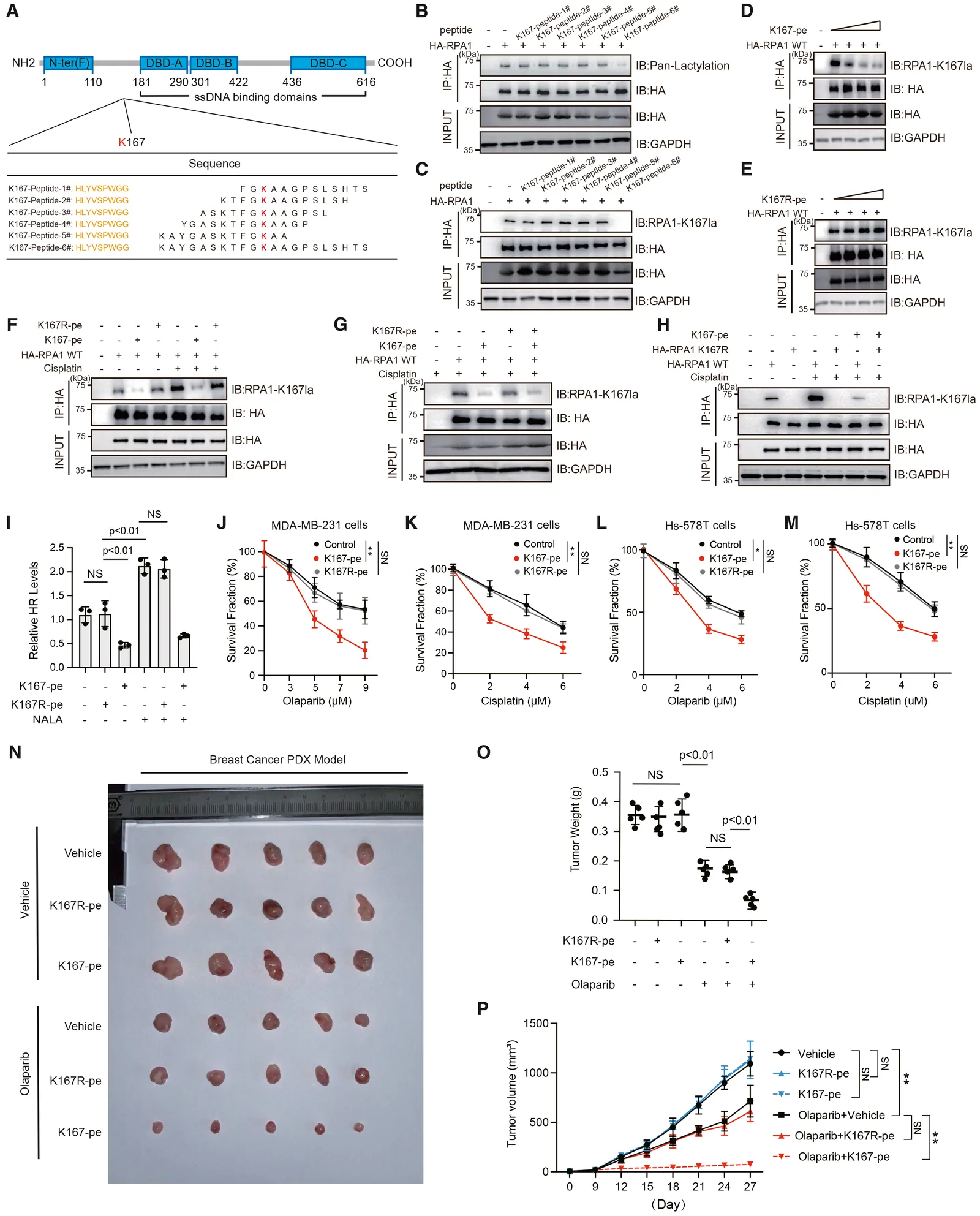
Peptidic inhibition of RPA1 K167 lactylation enhances chemosensitivity (A) Schematic illustration of designed peptides. Yellow: cell-penetrating peptide (CPP). Red: site for RPA1 lactylation. (B and C) Detection of HA-RPA1 lactylation in HEK293T cells treated with six different peptides using a pan-lactylation antibody (B) and a site-specific RPA1-K167la antibody (C). (D and E) Immunoblot analysis of RPA1 K167 lactylation in HEK293T cells treated with increasing concentrations of K167-pe (D) or K167R-pe (E). (F) Immunoblot analysis of RPA1 K167 lactylation in HEK293T cells expressing HA-RPA1 WT and treated with olaparib in the presence of K167-pe or K167R-pe. (G) Immunoblot analysis of RPA1 K167 lactylation in HEK293T cells expressing HA-RPA1 WT and treated with Cisplatin in the presence of K167-pe or K167R-pe. (H) Immunoblot analysis of RPA1 K167 lactylation in HEK293T cells expressing HA-RPA1 WT or HA-RPA1 K167R and treated with cisplatin in the presence or absence of K167-pe. (I) The HR levels in HEK293T cells treated with NALA, K167-pe, or K167R-pe were measured by FACS. (J–M) Cell viability of the MDA-MB-231 and HS-578T breast cancer cells treated with K167-pe or K167R-pe under olaparib (J and L) or cisplatin (K and M) stimulation. (N and O) Tumor images (N) from a breast cancer PDX model treated with vehicle, olaparib, K167-pe or K167R-pe, alone or in combination and quantification of tumor weight (O). (P) Tumor volume measurements over time in the breast cancer PDX model under different treatments. For plasmid transfection experiments, “−” indicates transfection with the corresponding empty vector, which was used as a negative control (B–H). Data are presented as mean ± SEM. Statistical significance was assessed by one-way ANOVA followed by Tukey’s multiple-comparison test (I and O) and two-way ANOVA (J–M and P). ∗*p* < 0.05; ∗∗*p* < 0.01; ∗∗∗*p* < 0.001; NS stands for no significant change.

Using the sequence of K167-pe, we synthesized a scrambled control peptide (K167R-pe) by replacing the lysine (K) at position 167 of K167-pe with arginine (R). We found that K167-pe inhibited RPA1 lactylation in a dose-dependent manner, whereas K167R-pe did not have an obvious inhibitory effect on RPA1 lactylation (Figures 6D and 6E). In addition, K167-pe, but not K167R-pe, significantly reduced RPA1 lactylation following DNA damage induced by cisplatin. (Figures 6F–6H). Furthermore, K167-pe treatment inhibited HR repair in HEK293T cells and increased the sensitivity of breast cancer cell lines to chemotherapeutic agents, whereas K167R-pe had no such effect (Figures 6I–6M).

We also confirmed these findings *in vivo* and found that K167-pe significantly increased the sensitivity of breast cancer PDX models to olaparib (Figures 6N–6P). Taken together, our results indicate that K167-pe inhibits RPA1 K167 lactylation, thereby compromising HR repair. This highlights K167-pe as a promising therapeutic approach for overcoming chemoresistance in breast cancer.

## Discussion

Cancer cells commonly exhibit a metabolic shift toward aerobic glycolysis—a phenomenon known as the Warburg effect—which generates excess lactate even under normoxic conditions.^57^^,^^58^^,^^59^ Although lactate was traditionally viewed as a waste product, it has recently been recognized as a key metabolic signal capable of mediating lysine lactylation on both histone and non-histone proteins.^33^ Lactate-mediated lactylation has been documented to exert pivotal regulatory roles in regulating cancer progression, including immune evasion and chemoresistance.^60^^,^^61^ Notably, MRE11 and NBS1—key components of the MRN complex, which is indispensable for initiating DNA end resection and HR repair—have been demonstrated to undergo lactylation, thereby promoting HR-mediated DNA repair and contributing to chemoresistance, suggesting that protein lactylation serves as a critical regulator of HR repair.

In this study, we demonstrate that RPA1, a key regulator of HR repair that binds and protects single-stranded DNA (ssDNA) generated after DNA end resection, represents a lactylation substrate at lysine 167 (K167), with KAT8 serving as the catalytic enzyme, in response to genotoxic stress. Furthermore, following DNA damage, KAT8 is phosphorylated by ATM at S392, a modification essential for the interaction between RPA1 and KAT8. Notably, lactylation of RPA1 at K167 enhances its chromatin loading and DNA-binding capacity, ultimately facilitating efficient HR repair and contributing to chemoresistance in cancer cells. Moreover, RPA1-K167R mutant, LDH inhibitors, or KAT8 inhibitors treatment downregulating RPA1 lactylation sensitizes cancer cells to chemotherapy. We further demonstrate a peptide (K167-pe) that selectively blocks RPA1 lactylation sensitized tumors to chemotherapy *in vitro* and in PDX models.

RPA is a central coordinator of the DDR and is extensively regulated by multiple PTMs. Among these, phosphorylation of the RPA2 subunit is one of the most extensively characterized modifications following DNA damage. Upon replication stress or DNA damage, DDR kinases such as ATR, ATM, and DNA-PK are activated and mediate multisite phosphorylation of RPA2, thereby contributing to replication stress signaling, DNA end resection, and HR repair.^62^^,^^63^ In addition to phosphorylation, both SUMOylation and acetylation of RPA have been reported to facilitate DNA repair. For example, DNA damage-induced SUMOylation of RPA1 promotes HR repair through facilitating the recruitment of HR repair factors, including RAD51.^64^ Collectively, these studies suggest that distinct PTMs form a complex regulatory network that fine-tunes RPA function to ensure efficient and coordinated DNA repair following genotoxic stress. Our findings further expand this regulatory landscape and reveal an important role for KAT8-mediated RPA1 lactylation in promoting RPA chromatin loading and HR repair following DNA damage. Notably, we developed a competitive peptide derived from the RPA1 K167 region (K167-pe) that interferes with RPA1 lactylation and sensitizes tumors to chemotherapy in both cultured cells and PDX models. These findings suggest that targeting RPA1 lactylation, rather than globally inhibiting RPA function, may represent a potentially feasible strategy for overcoming chemoresistance.

Previous studies have primarily linked KAT8 to DNA damage repair through its ability to remodel chromatin via H4K16 acetylation. KAT8-mediated H4K16 acetylation facilitates chromatin relaxation and promotes the recruitment of DNA repair factors to sites of DNA damage, thereby contributing to efficient DNA damage signaling and repair.^65^ More recently, KAT8 has also been identified as a lysine lactyltransferase, suggesting that its enzymatic activity is not limited to acetylation.^66^ However, whether KAT8 directly regulates core DNA repair machinery through lactylation has remained largely unknown. In the present study, we identify RPA1 as a direct non-histone lactylation substrate of KAT8 and demonstrate that ATM-dependent phosphorylation of KAT8 is required for efficient RPA1 lactylation following DNA damage. These findings uncover a previously unrecognized ATM-KAT8-RPA1 signaling axis and reveal a mechanism through which KAT8 regulates HR repair by directly modifying a core DNA repair factor. Thus, our study expands the functional landscape of KAT8 in the DDR and suggests that KAT8-mediated lactylation can serve as a direct regulatory mechanism for DNA repair. Notably, pharmacological inhibition of KAT8 reduced RPA1 lactylation and enhanced chemosensitivity in our experimental models. These findings suggest that KAT8-dependent lactylation may contribute to therapeutic resistance in cancer cells and highlight its potential therapeutic relevance.

Recent work has identified HAT1 as a lactyltransferase that mediates RPA1 K167 lactylation and promotes DNA repair and radioresistance in lung adenocarcinoma models.^67^ Consistent with these findings, we also identified K167 as a major lactylation site on RPA1, further supporting its functional importance in the DDR. In contrast to the study by He et al., which focused on HAT1-mediated RPA1 lactylation and radioresistance in lung adenocarcinoma, our study reveals an ATM-KAT8-RPA1 regulatory axis in which DNA damage-induced phosphorylation of KAT8 at S392 promotes its interaction with RPA1 and facilitates RPA1 K167 lactylation, thereby enhancing HR repair. Furthermore, we developed a CPP targeting RPA1 K167 lactylation and demonstrated its therapeutic efficacy in sensitizing breast cancer PDX tumors to chemotherapy. Together, these findings expand our understanding of the regulation and biological functions of RPA1 lactylation and highlight the therapeutic potential of targeting this modification to overcome chemoresistance.

LDHA and LDHB are two major LDH isoforms that jointly catalyze the reversible interconversion between pyruvate and lactate and play complementary roles in maintaining lactate metabolism and redox homeostasis in tumor cells.^68^^,^^69^ From a metabolic perspective, LDHA preferentially catalyzes the conversion of pyruvate to lactate and sustains glycolytic flux by regenerating NAD^+^, which is an important component of the Warburg effect in cancer cells.^68^^,^^70^ Upregulation of LDHA promotes lactate accumulation, acidification of the tumor microenvironment, and tumor progression, and is associated with poor prognosis in multiple cancer types.^68^^,^^70^ On the other hand, LDHB is more closely involved in lactate reutilization and mitochondrial oxidative metabolism by converting lactate to pyruvate, thereby allowing certain tumor cells to use lactate as an energy and carbon source under specific metabolic conditions.^68^^,^^71^ These established metabolic functions provide an important metabolic context for the mechanism proposed in this study. In addition, previous studies have shown that depletion of either LDHA or LDHB reduces intracellular lactate levels in breast cancer cells.^72^ Given the critical role of LDHA/B in maintaining intracellular lactate levels, we depleted LDHA/B to reduce intracellular lactate and further evaluated its effects on RPA1 lactylation and RPA1-mediated DNA repair.

The effects of KAT8i, LDHi, and K167-pe were more pronounced in the PDX model than in cultured cancer cells. One possible explanation is that PDX models preserve important features of the native tumor microenvironment and intratumoral heterogeneity, including stromal interactions and vascular architecture, which are largely absent in conventional cell culture systems and are thought to influence therapeutic responses.^73^ Whether these factors account for the enhanced efficacy observed in the PDX model remains to be further investigated.

In conclusion, our study identifies RPA1 lactylation at K167 a critical mechanistic node bridging tumor metabolic to HR proficiency and chemoresistance. Targeting this RPA1 lactylation may expand the therapeutic landscape for patients with HR-proficient tumors who are refractory to current DDR inhibitors.

### Limitations of the study

This study has several limitations. First, although we demonstrate that ATM-dependent phosphorylation of KAT8 promotes RPA1 lactylation following DNA damage, the structural basis underlying the interaction between phosphorylated KAT8 and RPA1 remains to be determined. Second, while targeting RPA1 lactylation enhanced chemosensitivity in cultured cancer cells and PDX models, the therapeutic efficacy and safety of this strategy require further evaluation in additional preclinical models and tumor types.

## Resource availability

### Lead contact

Further information and requests for resources and reagents should be directed to and will be fulfilled by the lead contact, Jian Yuan (yuan.jian80@outlook.com).

### Materials availability

All unique/stable reagents generated in this study are available from the lead contact without restriction.

### Data and code availability

• All data reported in this paper will be shared by the lead contact upon request.
• This paper does not report original code.
• Any additional information required to reanalyze the data reported in this paper is available from the lead contact upon request.

## Acknowledgments

This work was supported by the Startup fund of Shanghai East Hospital (DFRC2024011). We thank Xiaoyun Mao for providing the human breast cancer tissue samples.

## Author contributions

J.Y., conceptualization, funding acquisition, project administration, resources and writing – review and editing; X.D., data curation, formal analysis, investigation, validation and writing; J.Y., data analysis, validation and writing – review and editing; J.W., M.J., Z.W., X.Y., X.W., Y.S., B.H., R.L., Y.L., Y.H., W.Z., Q.W., Z.Z., validation and providing constructive suggestions; Y.C., conceptualization, validation and writing – review and editing.

## Declaration of interests

The authors declare no competing interests.

## STAR★Methods

### Key resources table

**Table:** 

REAGENT or RESOURCE	SOURCE	IDENTIFIER
**Antibodies**		

Anti-GAPDH	Proteintech	Cat# 60004-1-lg; RRID: AB_2107436
Anti-γH2AX	Millipore	Cat# 05-636; RRID: AB_309864
Anti-HA	Cell Signaling Technology	Cat# 3724; RRID: AB_1549585
Anti-Myc	Proteintech	Cat# 16286-1-AP; RRID: AB_11182162
Anti-RPA2	Santa Cruz Biotechinology	Cat# sc-56770; RRID: AB_785534
Anti-p-S4/8-RPA2	Thermo Fisher Scientific	Cat# A300-245; RRID: AB_210547
Anti-Pan-Lac	PTM Biolab	Cat# PTM-1401RM; RRID: AB_2942013
Anti-LDHA	Cell Signaling Technology	Cat# 2012; RRID: AB_2137173
Anti-LDHB	Thermo Fisher Scientific	Cat# PA5-100012; RRID: AB_2815542
Anti-H3	Proteintech	Cat# 17168-1-AP; RRID: AB_2716755
Anti-RPA1-K167la	ABclonal	N/A
Anti-IgG	Abmart	Cat# M21001; RRID: AB_2713950
Anti-Pan-Ace	Cell Signaling Technology	Cat# 9441; RRID: AB_331805
Anti-RAD51	GeneTex	Cat# GTX100469; RRID: AB_1951602
Anti-Flag	Sigma-Aldrich	Cat# F1804; RRID: AB_262044
Anti-Pan-Lac	PTM Biolab	Cat# PTM-1401RM; RRID: AB_2942013
Anti-ATM	Cell Signaling Technology	Cat# 2873; RRID: AB_2062659
Anti-Ki67	Cell Signaling Technology	Cat# 9449; RRID: AB_2797703
Anti-KAT8	Cell Signaling Technology	Cat# 46862; RRID: AB_2799311
Anti-p-SQ/TQ ATM/ATR Substrate	Cell Signaling Technology	Cat# 2851; RRID: AB_330318

**Bacterial and virus strains**		

BL21	TIANGEN	Cat# CB105
STBL3	Thermo Fisher Scientific	Cat# C737303
DH5α	TIANGEN	Cat# CB101

**Biological samples**		

Human breast cancer tissue	First Affiliated Hospital of China Medical University	N/A

**Chemicals, peptides, and recombinant proteins**		

Cisplatin	Sigma-Aldrich	Cat# P4394
Etoposide	Sigma-Aldrich	Cat# 341205
Bleomycin	Sigma-Aldrich	Cat# 203401
Penicillin/Streptomycin	GIBCO	Cat# 15140-122
Sodium L-lactate	Sigma-Aldrich	Cat# 71718
LDH inhibitor/Sodium oxamate	Sigma-Aldrich	Cat# O2751
KU55933/ATMi	Sigma-Aldrich	Cat# SML1109
PARP inhibitor/Olaparib	Selleckchem	Cat# AZD2281
RPA1-K167-Pe	Guoping Pharmaceutic	Inc,N/A
RPA1-K167R-Pe	Guoping Pharmaceutic	Inc,N/A
Polyethylenimine	Polysciences	Cat# 23966
Lipofectamine™ 2000	Thermo Fisher Scientific	Cat# 11668027
Lipofectamine™ 3000	Thermo Fisher Scientific	Cat# L3000150
Polybrene	Sigma-Aldrich	Cat# H9268
XhoI	New England Biolabs	Cat# R0146V
Micrococal nuclease	New England Biolabs	Cat# M0247S
DTT	Sigma-Aldrich	Cat# 3483-12-3
Puromycin	Sigma-Aldrich	Cat# 540222
Acetyl-CoA	Sigma-Aldrich	Cat# A2056
Isopropyl b-D-1-thiogalactopyranoside (IPTG)	Thermo Fisher Scientific	Cat# 15529019
Anti-Flag-agarose beads	Milipore	Cat# A2220
Anti-GST-agarose beads	Cytiva	Cat# 17075601
NP-40	Sigma-Aldrich	Cat# 127087-87-0
HEPES	Sigma-Aldrich	Cat# H3662
Sodium deoxycholate	Sigma-Aldrich	Cat# 30968
Anti-HA-agarose beads	Milipore	Cat# A209
Protein A/G -agarose beads	Milipore	Cat# IP10
Lactyl-CoA	MCE	Cat# HY-141540

**Critical commercial assays**		

Nuclear and Cytoplasmic Protein Extraction Kit	beyotime	P0028
Cell Counting Kit-8	beyotime	C0038
Plasmid Extraction Kit	TIANGEN	Cat# DP103

**Experimental models: Cell lines**		

Human: U2OS cells	ATCC	Cat# HTB-96
Human: HEK-293T cells	ATCC	Cat# CRL-3216
Human: MDA-MB-231 cells	ATCC	Cat# HTB-26
Human: SUM-159 cells	Generous gift of Dr. Chunlong Zhong	N/A
Human: HCC1806 cells	ATCC	Cat# CRL-2335
Human: HS578T cells	ATCC	Cat# HTB-126

**Experimental models: Organisms/strains**		

BALB/c Nude mice	Charles River	Strain# 213

**Oligonucleotides**		

See Table S1	This paper	N/A

**Recombinant DNA**		

pLVX-puro-HA-RPA1 WT	This paper	N/A
pLVX-puro-HA-RPA1 K167R	This paper	N/A
pLVX-puro-HA-CBP	This paper	N/A
pLVX-puro-HA-P300	This paper	N/A
pLVX-puro-Myc-KAT5	This paper	N/A
pLVX-puro- Myc-KAT8	This paper	N/A
pLVX-puro- Myc-PCAF	This paper	N/A
pLVX-puro- Myc-GCN5	This paper	N/A

**Software and algorithms**		

Graphpad Prism (version 8.4.0)	Graphpad Softwar	https://www.graphpad.com/
ImageJ (version 1.5.2)	BD Life Sciences	https://www.flowjo.com/
Adobe Illustrator	Adobe	https://www.adobe.com/hk_zh/products/illustrator/free-trial-download.html
FlowJo software (version 10.8.1)	BD Life Sciences	https://www.flowjo.com/

### Experimental model and study participant details

#### Animal

Six-week-old female BALB/c nude (nu/nu) mice were used in this study. Mice were housed under specific pathogen-free (SPF) conditions at 25°C and 50% relative humidity with a 12-h light/dark cycle and *ad libitum* access to food and water. Female mice were selected to ensure consistency in the experimental conditions across the *in vivo* studies. All animal studies were conducted in accordance with the Guide for the Care and Use of Laboratory Animals (National Academies Press, 2011), as mandated by the U.S. National Institutes of Health. All procedures were reviewed and approved by the Ethics Committee of Tongji University School of Medicine (approval number: TJBB02524110) and carried out under institutional guidelines for animal welfare. As only female mice were used in the experiments, potential sex-dependent differences were not assessed.

#### Patient-derived xenograft (PDX) models

Breast tumor tissue used to establish the patient-derived xenograft (PDX) model was obtained from surgical resection at the First Affiliated Hospital of China Medical University. Human tissue was obtained from a 63-year-old female patient with grade G3 breast cancer and TNM stage 320, with Ki67 (+), CerbB-2 (−), ER (−), and PR (−). HR repair functionality was wild-type at the time of tissue collection. All human tissue collection procedures were approved by the Ethics Committee of the First Hospital of China Medical University and were conducted in accordance with institutional guidelines and the Declaration of Helsinki (Approval No. AF-SOP-07-1.2-01). Approximately 8 mm^3^ of tumor tissue was implanted subcutaneously into 6-week-old female BALB/c nude mice to establish the PDX model. The PDX model used in this study was derived from tumor tissue obtained from one patient. The patient-derived tumor tissue was initially propagated in female BALB/c nude mice. Once the xenograft tumors reached a sufficient size, tumors were excised and divided into approximately equal-sized fragments (∼8 mm^3^), which were subsequently implanted subcutaneously into 6-week-old female BALB/c nude mice for subsequent PDX experiments.

#### Cells

Cell lines including HEK293T, U2OS, MCF10A, MDA-MB-231, HCC1806, and Hs-578T were obtained from the American Type Culture Collection (ATCC). SUM-159-PT cells were kindly provided by Dr. Chunlong Zhong. Unless otherwise indicated, cells were cultured in Dulbecco’s modified Eagle medium (DMEM) supplemented with 10% fetal bovine serum (FBS) and maintained at 37°C in a humidified atmosphere containing 5% CO_2_. All cell lines were routinely tested for mycoplasma contamination and were confirmed to be mycoplasma-free. No additional cell line authentication was performed in our laboratory.

### Method details

#### In vivo sodium lactate administration

In animal experiments, mice received daily intraperitoneal injections of sodium lactate at a dosage of 120 mg/kg.

#### Mass spectrometry analysis

To identify lactylation sites on RPA1, we analyzed a previously published lactylome dataset available in the PRIDE database (PRIDE: PXD055229; PRIDE: PXD055225). In the original study, HEK293T cells were exposed to 10 Gy irradiation and harvested 2 h later. Cellular proteins were extracted and subjected to tryptic digestion, followed by affinity enrichment of lysine lactylation (Kla)-containing peptides. The enriched peptides were separated using an EASY-nLC 1000 UPLC system and analyzed on a Q Exactive Plus mass spectrometer (Thermo Fisher Scientific). MS/MS spectra were processed using MaxQuant (v1.5.2.8) against the corresponding human protein database with a reverse decoy database. Trypsin/P was specified as the proteolytic enzyme with up to four missed cleavages. The precursor mass tolerances were set to 20 ppm for the first search and 5 ppm for the main search, whereas the fragment mass tolerance was 0.02 Da. Carbamidomethylation of cysteine was specified as a fixed modification, while methionine oxidation and lysine lactylation were considered variable modifications. The false discovery rate (FDR) was controlled at <1%. The processed lactylome dataset was subsequently queried for RPA1-derived Kla-containing peptides to identify candidate lactylation sites on RPA1.

#### Intestinal villi regeneration assay

In C57BL/6 wild-type mice (8–12 weeks old, 5 animals per group), intraperitoneal pre-treatment was administered with LDH inhibitor (sodium oxamate, 1000 mg/kg, every other day), KAT8 inhibitor (15 mg/kg, every other day), or sodium lactate (120 mg/kg, daily), either individually or in combination, starting 5 days prior to whole-body irradiation at a dose of 10 Gy. Mice were housed in a specific pathogen-free (SPF) environment throughout the study. Post-irradiation, treatments were continued for an additional 5 days before sacrifice. Small intestinal tissues were collected for histological examination.

For LDHA knockout mice (8–12 weeks old, 5 animals per group), sodium lactate (120 mg/kg/day) or vehicle was administered via intraperitoneal injection for 5 consecutive days prior to whole-body irradiation at 6 Gy. Mice were maintained in an SPF environment and received the same treatment for 5 more days following irradiation before being euthanized. Small intestines were harvested for subsequent histological analysis.

#### PDX tumor treatment

Breast tissue specimens in this study were obtained from surgical resections at the First Affiliated Hospital of China Medical University. For subsequent mouse xenograft experiments, approximately 8 mm^3^ of tissue was implanted subcutaneously into 6-week-old female athymic nude (nu/nu) mice. Once the tumor volumes reached a range of 150–200 mm^3^, mice were randomly assigned to one of six treatment arms: (1) saline as a control, (2) sodium oxamate at 1000 mg/kg, (3) KAT8 inhibitor at 15 mg/kg, (4) Olaparib at 50 mg/kg, (5) a combination of Olaparib (50 mg/kg) and sodium oxamate (1000 mg/kg), and (6) a combination of Olaparib (50 mg/kg) with KAT8 inhibitor (15 mg/kg). All agents were administered intraperitoneally every other day, and tumor tissues were collected for analysis after 30 days. Tumor measurements were recorded throughout the study, following procedures consistent with previously described xenograft tumor assays.

In a parallel study investigating the synergistic effects of K167-peptides or their mutant variant K167R-peptides in conjunction with Olaparib in treating breast cancer PDXs, mice bearing tumors of 150–200 mm^3^ were also divided into six groups: (1) saline control, (2) K167R-pe administered at 5 mg/kg, (3) K167-pe at the same dosage, (4) Olaparib at 50 mg/kg, (5) Olaparib combined with K167R-pe (50 mg/kg + 5 mg/kg), and (6) Olaparib combined with K167-pe (50 mg/kg + 5 mg/kg). K167-pe and its mutant form were given once daily via intraperitoneal injection, while Olaparib was delivered on a thrice-weekly schedule through the same route.

#### Cell culture and treatment

Cell lines including HEK293T, U2OS, MCF10A, MDA-MB-231, HCC1806, and Hs-578T were obtained from the American Type Culture Collection (ATCC). SUM-159-PT cells were kindly provided by Dr. Chunlong Zhong. Unless indicated otherwise, the cells were propagated in Dulbecco’s Modified Eagle Medium (DMEM) containing 10% fetal bovine serum (FBS), maintained at 37°C under a humidified atmosphere with 5% carbon dioxide.

For protein analysis via western blotting, cells were commonly subjected to treatment with 20 μM Cisplatin, 10 μM of the KAT8 inhibitor (KAT8i), 20 μM synthetic peptides mimicking RPA1 K167 lactylation, 10 mM or 20 mM of the LDH inhibitor sodium oxamate (LDHi), and sodium lactate (NALA) at concentrations of 10 mM, 25 mM, or 50 mM.

In immunofluorescence-based experiments, typical treatment regimens included 10 μM Cisplatin, 10 μM KAT8i, 20 μM RPA1 K167 lactylation-mimicking peptides, 10 mM LDHi, and 25 mM NALA.

For colony formation analysis, standard treatments involved 5 μM KAT8i, 10 mM LDHi, and NALA at either 10 mM or 25 mM concentrations.

#### CCK-8 assay

Approximately 3000–4000 cells were seeded into each well of a 96-well plate, with three replicates per group. After 24 h, cells were treated with Vehicle, K167-pe, or K167R-pe. Following another 12 h of treatment, cells were exposed to increasing concentrations of Cisplatin or Olaparib. After 24 h, the supernatant was removed, and 10% CCK-8 reagent was added to each well. After 1-h incubation, absorbance at 450 nm was measured to assess cell viability.

#### DNA transfection, lentiviral packaging, and infection

Cells underwent transfection using either polyetherimide (PEI) or Lipofectamine 2000/3000 (Thermo Fisher Scientific), selected based on experimental conditions. For lentiviral packaging, expression constructs and helper plasmids (pMD2.G and psPAX2) were co-transfected, and culture supernatants were collected at 24-h and 48-h intervals post-transfection.

The resulting viral particles were applied to target cells along with polybrene at a final concentration of 8 μg/mL to enhance transduction efficiency. Following infection, cells were subjected to puromycin selection at 2 μg/mL in the culture medium to generate stable populations. The expression of introduced constructs in the selected clones was validated by western blotting.

#### Homologous recombination reporter assay

To evaluate the efficiency of DNA double-strand break (DSB) repair via homologous recombination, the DR-GFP reporter assay system was utilized. HEK293T cells, either stably expressing specific short hairpin RNAs (shRNAs) or in their unmodified form, were plated in triplicate in six-well culture dishes. After a 24-h incubation period, the cells were transfected with a DNA complex composed of 500 ng DR-GFP plasmid, 500 ng I-SceI endonuclease vector, 100 ng mCherry reporter, and 3 μL of Polyetherimide (PEI) as the transfection reagent.

Following transfection, cells were exposed to the appropriate chemical treatments after 24 h. At 48 h post-transfection, the cells were collected and analyzed using flow cytometry (FACS). Sorting was performed based on RFP (mCherry) positivity or dual positivity for both RFP and GFP signals. Quantification of homologous recombination efficiency was determined by calculating the proportion of double-positive cells, following previously established methodologies.

#### Immunofluorescence assay

Twenty-four hours ahead of the treatment procedure, cells were seeded onto six-well dishes equipped with sterile coverslips. Cells were rinsed twice with phosphate-buffered saline (PBS), followed by fixation in 4% paraformaldehyde (PFA) at ambient temperature for 15 min. Subsequently, the cells were permeabilized for 10 min using 1% Triton X-100.Prior to immunostaining, nonspecific binding sites were blocked by incubating the cells with 2% goat serum for 1 h at room temperature. Thereafter, primary antibodies were applied and left to incubate overnight at 4°C.Following three washes with PBS, cells were treated with fluorophore-conjugated secondary antibodies for 1 h at room temperature. After another set of three PBS washes, nuclear staining was performed using DAPI, incubated for approximately 2 min under the same temperature conditions.

Finally, cells were washed once more, mounted using an anti-fade reagent, and examined using a fluorescence microscope.

#### Co-immunoprecipitation and western blotting

For co-immunoprecipitation experiments involving transient expression, HEK293T cells were transfected with plasmids encoding either HA-tagged RPA1 or Myc-tagged KAT8. Following transfection, cells were lysed on ice for 30 min using NETN buffer (composed of 20 mM Tris-HCl at pH 8.0, 100 mM NaCl, 1 mM EDTA, and 0.5% Nonidet P-40), supplemented with a protease inhibitor cocktail.Cellular debris was removed by centrifugation at 12,000 rpm for 10 min, and the resulting supernatant containing soluble proteins was incubated with HA-conjugated agarose beads for 2 h at 4°C. The beads were subsequently washed three times with NETN lysis buffer to reduce nonspecific binding.To elute bound proteins, beads were boiled for 5 min in SDS sample buffer (1×), after which protein samples were separated using SDS–polyacrylamide gel electrophoresis (SDS–PAGE). Following electrophoresis, membranes were blocked with non-fat milk dissolved in TBST buffer and then probed with the appropriate primary antibodies as specified.

#### *In vitro* phosphorylation assay

*In vitro* phosphorylation reactions were performed using purified recombinant His-KAT8 and commercially purified active human ATM protein (14-933, Sigma). Purified His-KAT8 and ATM were mixed in a total reaction volume of 20 μL. The final reaction buffer contained 25 mM Tris-HCl (pH 7.5), 5 mM β-glycerophosphate, 2 mM DTT, 0.1 mM NaVO_4_, 10 mM MgCl_2_, 1 mM EGTA, 0.4 mM EDTA, and 0.5 mM ATP. The reaction was incubated at 30°C for 30 min. After incubation, SDS-PAGE loading buffer was added to stop the reaction and elute the proteins, and protein phosphorylation levels were analyzed by immunoblotting.

#### *In vitro* pull-down assay for protein interaction between RPA1 and KAT8

*In vitro* pull-down assays were performed to validate the direct interaction between KAT8 and RPA1 using purified recombinant proteins. Briefly, equal amounts of Anti-GST agarose beads were incubated with either GST-tagged RPA1 or GST (prepared in our laboratory using the pGEX vector system in Escherichia coli BL21) as a negative control in pull-down buffer containing 20 mM Tris-HCl pH 7.5, 150 mM NaCl, 0.1% NP-40, 10% glycerol, and protease inhibitors at 4°C for 2 h to allow immobilization. After washing three times with pull-down buffer to remove unbound proteins, the beads were subsequently incubated with His-KAT8 protein (VKEY-BIO, P2HI0024S) in the same buffer at 4°C for an additional 3 h. Following extensive washing with pull-down buffer to remove nonspecifically bound proteins, the bound complexes were eluted by boiling in SDS loading buffer. The eluates were then subjected to SDS-PAGE and analyzed by Western blotting using antibodies specific for KAT8 to detect its presence in the HA-RPA1 precipitates, thereby verifying the direct interaction between KAT8 and RPA1.

#### Extraction of soluble and chromatin-associated fractions

To isolate soluble protein fractions, cells were initially disrupted on ice using NETN buffer (composed of 20 mM Tris-HCl at pH 8.0, 100 mM NaCl, 1 mM EDTA, and 0.5% Nonidet P-40), supplemented with a complete protease inhibitor cocktail (×13), and incubated for 10 min. The lysates were subjected to centrifugation at 12,000 RPM for 10 min at 4°C, after which the supernatant was retrieved and designated as the total cellular extract. The residual pellet underwent three sequential washes with PBS to remove soluble components and was subsequently resuspended in chilled EBC2 buffer (50 mM Tris-HCl, pH 7.5, 300 mM NaCl, 5 mM CaCl_2_), followed by the addition of micrococcal nuclease (10 units). After sonication and an additional centrifugation under the same conditions, the resulting supernatant was collected and defined as the chromatin-enriched fraction.

#### Colony formation assay

A total of 1000–2000 cells, as indicated, were plated in triplicate into individual wells of standard six-well culture plates. For drug sensitivity evaluation, cells were allowed to adhere for 24 h prior to treatment with the specified chemotherapeutic agents at the designated concentrations. Following drug exposure, cultures were maintained under standard incubation conditions for 10 to 14 days to permit colony development. Formed colonies were subsequently stained using Giemsa solution and counted manually or using imaging software. Final results were adjusted based on the respective plating efficiencies to account for differences in initial cell viability.

#### In vitro lactylation assay

Recombinant GST-tagged RPA1 proteins were incubated with Myc-tagged KAT8 proteins, the latter purified from HEK293T cells, in a reaction mixture consisting of 50 mM HEPES (pH 7.8), 30 mM KCl, 0.25 mM EDTA, 5 mM MgCl_2_, 5 mM sodium butyrate, and 2.5 mM DTT, supplemented with 20 mM lactyl-CoA. The reactions were carried out at 30°C for 30 min to allow lactylation. Subsequently, 5× SDS sample buffer was added to terminate the reactions, followed by heat denaturation at 100°C for 5 min. Proteins were resolved by SDS-PAGE and analyzed by immunoblotting using the indicated antibodies.

#### Peptide synthesis

Peptides utilized in this study were synthesized by Guoping Pharmaceutical Co., Ltd. (Hefei, China). Prior to experimental application, all peptides underwent purification via high-performance liquid chromatography, ensuring a purity greater than 98%. For *in vivo* studies, peptides were composed entirely of D-amino acid residues to enhance stability. In preparation for *in vitro* assays, peptides were dissolved in phosphate-buffered saline (PBS) to yield stock solutions at a concentration of 10 mM. In contrast, K167-pe and K167R-pe, designated for *in vivo* administration, were freshly prepared in PBS, stored on ice, and equilibrated to room temperature immediately before injection.

### Quantification and statistical analysis

Quantitative data from cell viability assays and immunofluorescence-based foci analyses were obtained from at least three biologically independent experiments (*n* ≥ 3), and are presented as mean ± SEM. RPA1 foci were quantified using ImageJ (version 2.14.0). Cell nuclei were outlined as regions of interest (ROIs) using the Freehand Selections tool. Background subtraction was performed using a uniform rolling ball radius of 30 pixels. Within each ROI, foci were detected using the Find Maxima function with a prominence threshold of 50 to distinguish discrete foci from background. Only foci above this threshold were counted, and the number of RPA1 foci per nucleus was recorded.

For *in vivo* experiments, five mice were included in each experimental group (*n* = 5 mice per group), unless otherwise indicated, and data are presented as mean ± SEM. Statistical analyses were performed using GraphPad Prism software. Comparisons between two groups were performed using an unpaired two-tailed Student’s *t* test. For comparisons involving more than two groups, statistical significance was assessed using one-way ANOVA followed by Tukey’s multiple-comparison test or two-way ANOVA, as appropriate for the experimental design. *p* < 0.05 was considered statistically significant.
