# Supplementary material for: Metabolic regulation of ATM-KAT8-RPA1 axis in DNA damage repair and chemoresistance
Source: iScience. 2026 Oct 26;29(11):117908. doi: 10.1016/j.isci.2026.117908 (PMC13734786; doi:10.1016/j.isci.2026.117908)
Supplement: Supplementary file 1 [file mmc1.pdf]

iScience, Volume *29*

Supplemental information

Metabolic regulation of ATM-KAT8-RPA1

axis in DNA damage repair and chemoresistance

Xin Ding, Jie Yang, Jinhuan Wu, Mingpeng Jin, Zhe Wang, Xiaoning Yang, Xuanhe Wang, Yifei Song, Bingsong Huang, Rui Li, Yu Li, Yiming He, Wen Zheng, Qianwen Wang, Zhikai Zeng, Yuping Chen, and Jian Yuan

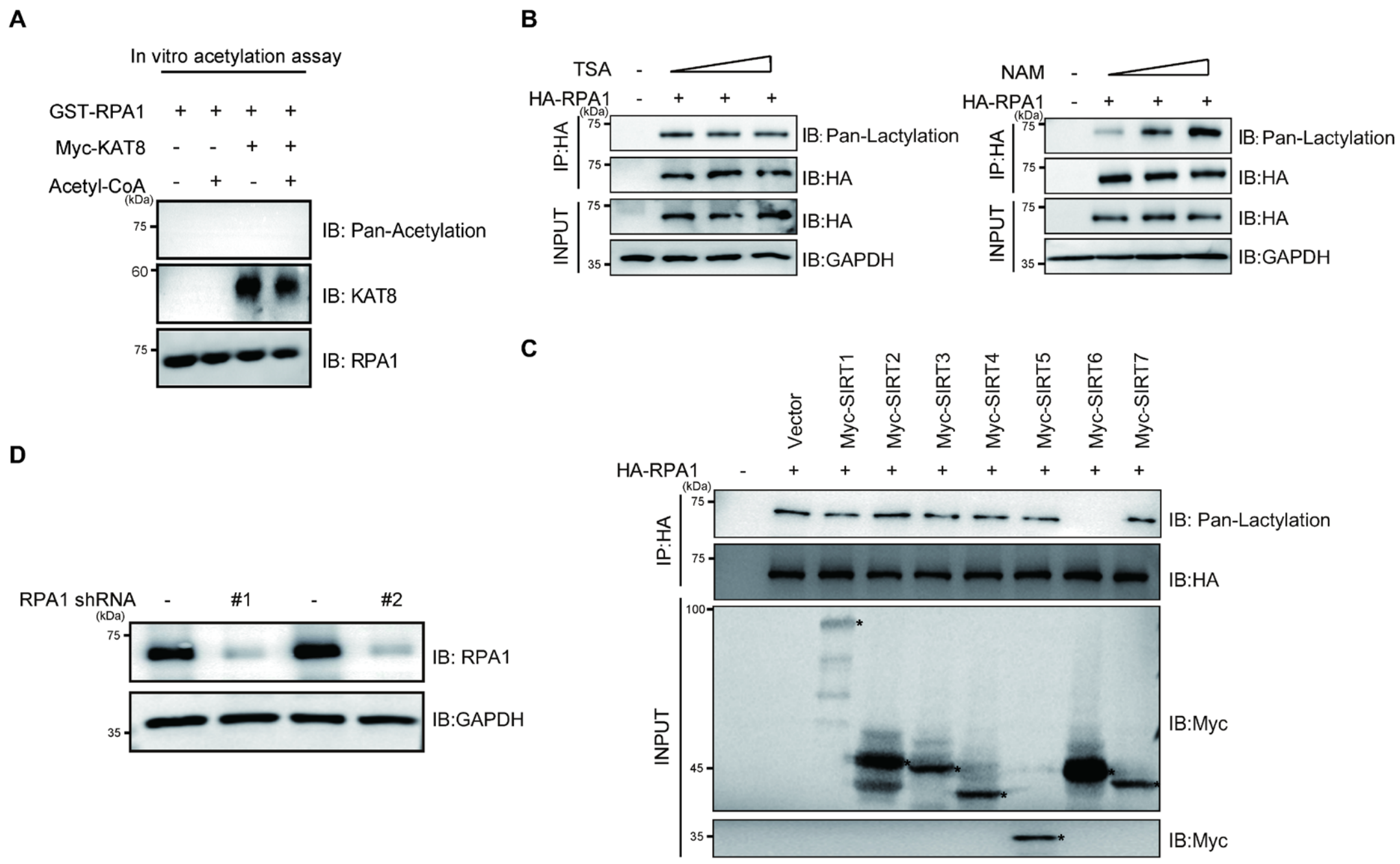

Figure S1. RPA1 is lactylated by KAT8 in response to DNA damage, Related to Figure 1

A. Detecting RPA1 acetylation using an in vitro acetylation assay on the indicated samples.

B. Immunoblot analysis of HA-RPA1 lactylation in HEK293T cells treated with increasing concentrations of TSA（0 μM, 5 μM and 10 μM) or NAM（0 mM, 5 mM and 10 mM).

C. Immunoblot analysis showing that SIRT6 overexpression specifically reduces HA-RPA1 lactylation in HEK293T cells.

D. Immunoblot analysis showing knockdown efficiency of RPA1 shRNAs in HEK293T cells.

For plasmid transfection experiments, "–" indicates transfection with the corresponding empty vector, which was used as a negative control (A-C).

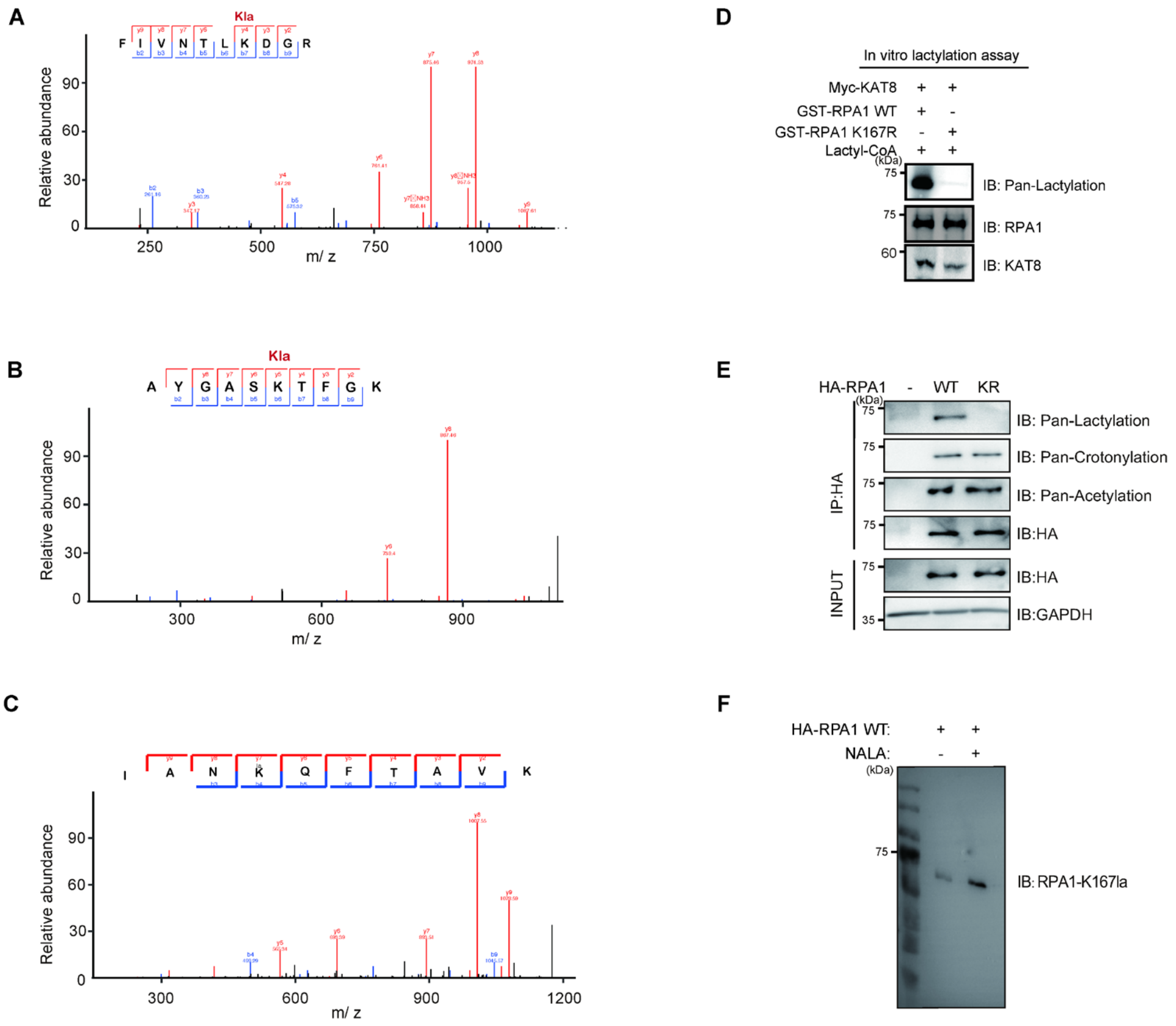

Figure S2. RPA1 is modified by lactylation at K167, Related to Figure 2

A-C. Illustration of RPA1 K88, K163 and K267 lactylation identified by MS.

D. In vitro lactylation assay showing reduced lactylation of GST-RPA1 K167R compared with GST-RPA1 WT in the presence of KAT8 and lactyl-CoA.

E. Immunoblot analysis of lactylation, crotonylation and acetylation of HA-RPA1 WT and HA-RPA1 K167R in HEK293T cells.

F. Validation of the RPA1-K167la-specific antibody by immunoblot analysis in lysates from HA-RPA1 WT-expressing HEK293T cells treated with or without Sodium lactate (NALA).

For plasmid transfection experiments, "–" indicates transfection with the corresponding empty vector, which was used as a negative control (E).

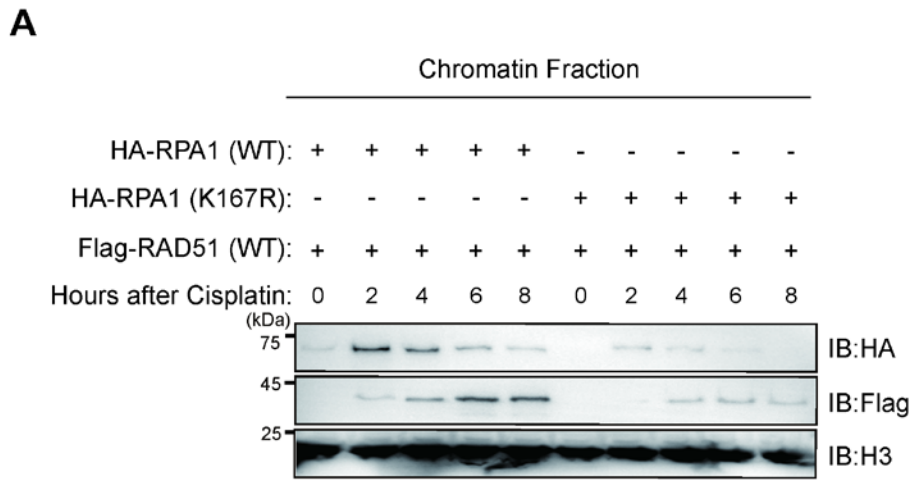

Figure S3. RPA1 K167 lactylation facilitates chromatin recruitment of RPA1 and RAD51, Related to Figure 3

A. Chromatin fractionation analysis of HA-RPA1 WT or HA-RPA1 K167R together with Flag-RAD51 in HEK293T cells at the indicated time points following Cisplatin treatment.

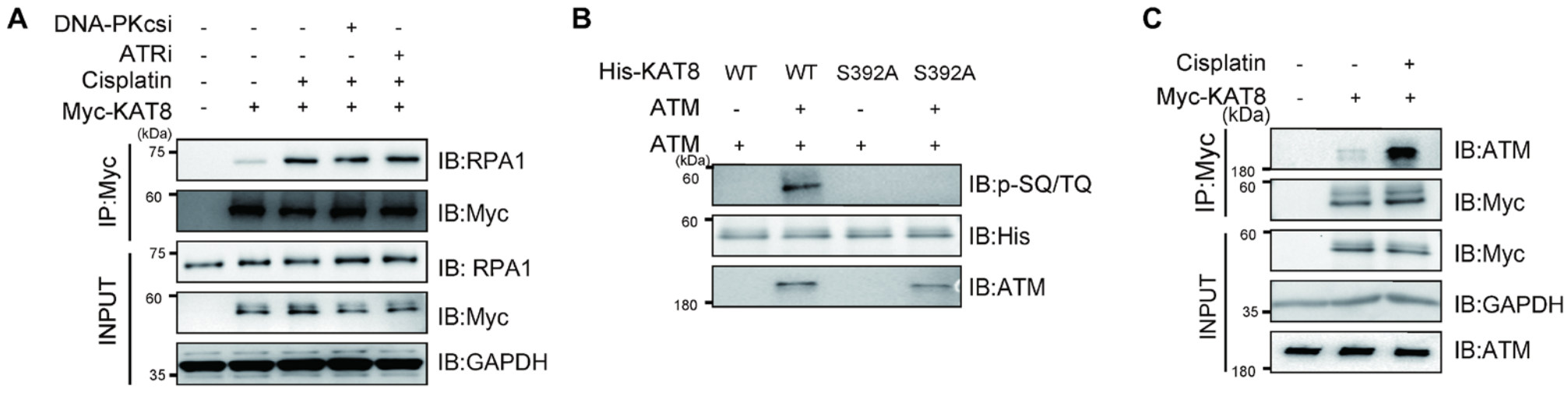

Figure S4. ATM-mediated phosphorylation of KAT8 promoted RPA1 foci formation upon DNA damage, Related to Figure 4

A. Co-immunoprecipitation analysis of the interaction between Myc-KAT8 and endogenous RPA1 in HEK293T cells treated with Cisplatin in the presence or absence of KU-57788 (DNA-PKcs inhibitor/ DNA-PKcsi) or Ceralasertib (ATR inhibitor/ ATRi).

B. In vitro kinase assay showing ATM-mediated phosphorylation of His-KAT8 WT but not the His-KAT8 S392A mutant.

C. Co-immunoprecipitation analysis of the interaction between Myc-KAT8 and endogenous ATM in HEK293T cells following Cisplatin treatment.

For plasmid transfection experiments, "–" indicates transfection with the corresponding empty vector, which was used as a negative control (A and C).

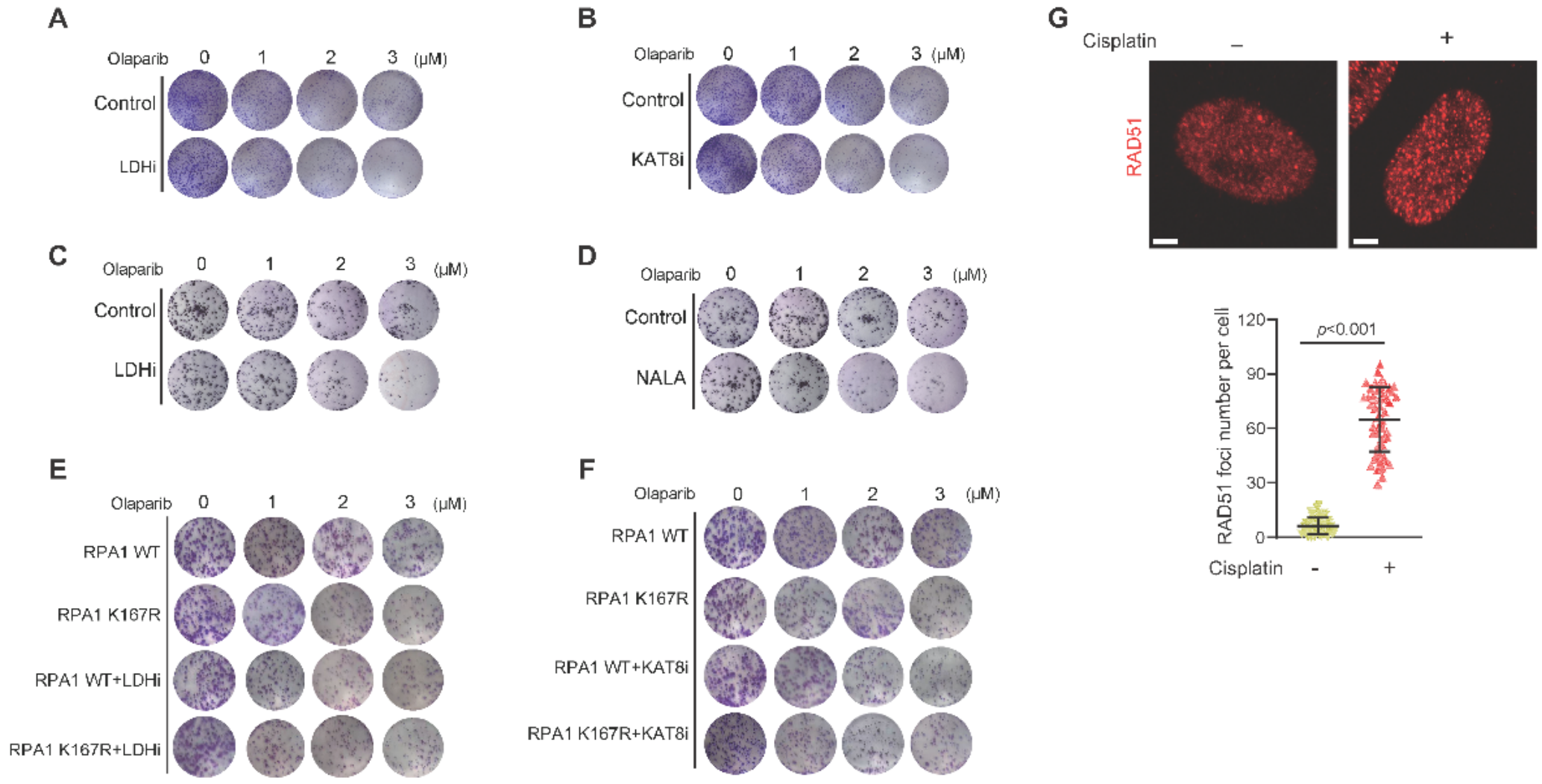

Figure S5. The breast cancer patient-derived xenograft model is homologous recombination proficient, Related to Figure 5

A. Representative immunofluorescence images of RAD51 foci formation in PDX-derived tumor cells following Cisplatin treatment. Scare bar, 4 μm.

Data are presented as mean ± SEM. Statistical significance was assessed by unpaired Student's t test (B). ***$p < 0.001$.

Table S1. Sequences used in this study, Related to STAR Methods

shLDHA-1	5'GCTGGGAGTTCACCCATTAAGTTCAAGAGACTTAATGGGTGAACTCCCAGCT TTTTTG 3'
shLDHA-2	:5'GGAACAGTGGAAAGAGGTTCATTCAAGAGATGAACCTCTTTCCACTGTTCCT TTTTTG 3'
shLDHB-1	5'GGAAGAAGAGGCAACAGTTCCTTCAAGAGAGGAACTGTTGCCTCTTCTTCCT TTTTTG 3'
shLDHB-2	:5'CGTGATTGGAAGTGGATGTAATTCAAGAGATTACATCCACTTCCAATCACGT TTTTTG 3'
shKAT8-1	5'-GCAAGATCACTCGCAACCAAA-3'
shKAT8-2	5'-CGAAATTGATGCCTGGTATTT-3'
